# Targeted digital voter suppression efforts likely decrease voter turnout

**DOI:** 10.1073/pnas.2519944123

**Published:** 2026-01-26

**Authors:** Young Mie Kim, Ross Dahlke, Hyebin Song, Richard Heinrich

**Affiliations:** ^a^The School of Journalism and Mass Communication and the Department of Political Science (Faculty Affiliate), University of Wisconsin, Madison, WI 53706; ^b^The School of Journalism and Mass Communication, University of Wisconsin, Madison, WI 53706; ^c^The Department of Statistics, Pennsylvania State University, University Park, PA 16802; ^d^Senior Consumer Insights Strategist, The Wisconsin School of Business, University of Wisconsin, Madison, WI 53706

**Keywords:** voter suppression, foreign election interference, social media, microtargeting, advertising

## Abstract

In light of continued foreign interference in the US presidential elections, where undisclosed digital voter suppression advertising has been deployed, this study addresses the questions of who is exposed to these ads and whether and how such exposure influences voter turnout. Using a sample that resembles the US voting-age population, the study directly measures each individual’s ad exposure through a user-level real-time ad tracking tool, which is merged with the same individual’s survey responses to identify voter suppression content and its targeting patterns. By further matching individual-level exposure to voter suppression ads with the same individual’s verified voter turnout records, the study estimates the effects of voter suppression on actual turnout. The study findings from the 2016 US Presidential Election reveal clear geo-racial targeting patterns in voter suppression: non-Whites residing in the racial minority counties of battleground states were exposed to substantially more voter suppression ads than their counterparts. Moreover, exposure to voter suppression ads was associated with decreases in voter turnout at the population level, albeit small. The sharpest declines were observed among non-Whites residing in minority counties of battleground states, suggesting that the intensified turnout suppression among the targeted segments of the electorate may have played a role in shaping turnout.

## Significance

Although governments have repeatedly flagged digital voter suppression in recent elections, such reports have remained anecdotal. Using a sample that mirrors the US voting-age population, this study provides first systematic empirical documentation of undisclosed digital voter suppression, including foreign election interference, based on the case of the 2016 US Presidential Election. By tracking each user’s digital ad exposure, demographic profiles, and verified voting records, this study reveals that digital voter suppression disproportionally targeted non-Whites in racial minority counties of battleground states. Individuals exposed to digital voter suppression ads were less likely to vote. The findings offer real-world evidence that targeted digital voter suppression may decrease voter turnout, raising serious concerns about election integrity in data-driven, microtargeted information environments.

Just months ahead of the 2024 Elections, the US Department of Justice (DoJ) seized numerous digital outlets as part of Russia’s ongoing election interference (1). The operation implemented various tactics designed to suppress voter turnout in swing states, such as disseminating false information about voting, attacking particular candidates, and even orchestrating bomb threats at polling stations (2).

**Fig. 1.**
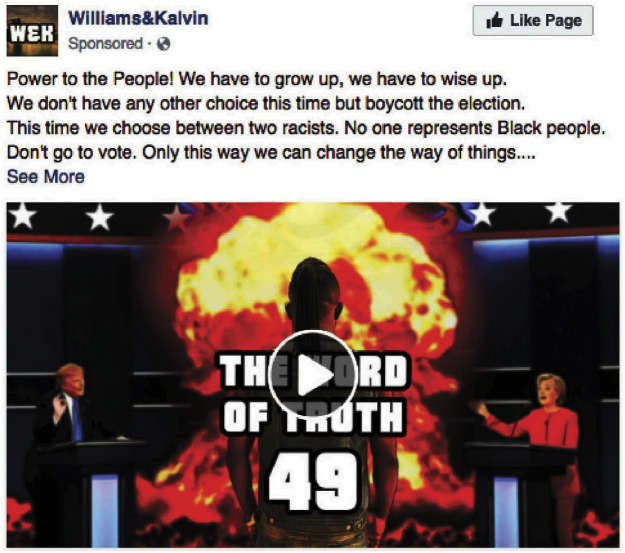
An example of voter suppression ads on Facebook: Don’t Go to Vote. This ad later turned out to be part of the Russian Election Interference Campaigns. Also available at the US House of Representatives Permanent Select Committee on Intelligence, “Social Media Content” advertisement archive (AD 3026) for “Exposing Russia’s Effort to Sow Discord Online: The Internet Research Agency and Advertisements.”

These efforts follow the same strategies Russia employed in the 2016 US Elections. According to the bipartisan Senate Intelligence Report (3), the Internet Research Agency (IRA)—a Russian digital disinformation campaign operation—promoted specific political agendas while suppressing the turnout from non-White voters. For example, one of its Facebook ads (Fig. 1) (4), posted under the guise of an African American group, reads, “…We don’t have any other choice this time but boycott the election. … No one represents Black people. Don’t go to vote. …” The IRA was not the only entity operating targeted voter suppression. Disguised as a Hillary Clinton campaign, a Florida man ran a social media campaign titled “Avoid the line. Vote from home,” specifically targeting non-Whites—an effort that ultimately resulted in a federal conviction for conspiring to deprive individuals of their right to vote (5). In the 2020 US elections, as another instance, voters in battleground states received phone calls saying, “Stay safe, stay home” on election day (6).

Voter suppression—a strategy that demobilizes, discourages, or prevents specific segments of the population from voting (7)—has persisted since the post-Civil War and Reconstruction eras (8). Historically, it has taken many forms, ranging from blatant violence, intimidation, or threat to regulatory devices such as voter ID laws and gerrymandering, as well as flyers containing misinformation about the time, manner, or place of voting (9). Targeted voter suppression has recently moved to the digital sphere, capitalizing on its microtargeting capacity (10, 11). The meta information associated with the voter suppression ad, “… Don’t go to vote. …” (Fig. 1)—submitted to the US House Permanent Select Committee on Intelligence by Meta (formerly Facebook)—indicates that the IRA purchased Facebook ads using the platform’s Detailed Interest Targeting method. It used Facebook’s preset interest terms, such as “Martin Luther King, Jr.,” “African American Civil Rights Movement,” “African American history,” “Malcolm X,” and “United States presidential election 2016,” to target African American voters specifically (4). With its invisible microtargeting, targeted digital voter suppression thrives on social media as undisclosed campaigns—advertising campaigns that do not file reports with the Federal Election Commission (FEC) or the Internal Revenue Service (IRS) (11). The absence of conspicuous disclosure and disclaimer rules on digital campaigns, coupled with the Supreme Court’s *Citizens United* (12) decision permitting unlimited campaigns by anonymous sponsors, has opened the door for voter suppression by undisclosed campaigns to proliferate online without revealing their true identities (13). Voter suppression in the digital age thus poses consequential threats to democracy.

Despite broad normative concern, little scientific research independent of governments or digital platform companies has investigated digital voter suppression by undisclosed campaigns. Who is targeted and exposed to digital voter suppression? Does exposure to targeted digital voter suppression have any impact on voter turnout, especially among targeted voters? Using independently collected individual user-level exposure data and linked to actual individual-level voter turnout records, this study empirically and systematically addresses those research questions.

Research on social media has increased exponentially over the past few years, including notable large-scale randomized controlled trials by the Meta-Academics Initiative (14–16). However, research in general has paid relatively less attention to targeted digital campaigns. Furthermore, despite the pervasive adoption of microtargeting in political campaigns (17) and a great deal of heterogeneity observed in users’ platform behavior (18), research on digital platforms still primarily focuses on average treatment effects (ATEs), while often neglecting differential effects across targeted subpopulations. Recent studies on European elections indicate that political campaigns have adopted a wide range of targeted advertising strategies using social media, including demographic, geographic, issue-based targeting, and coalition-breaking strategies, suggesting potential differential effects of targeted advertising. However, when focusing on the ATEs, the previous studies often found “null” results—results without expected outcomes—implying an inconclusive effect of social media. In contrast, another recent large-scale field experiment reported that while no ATE of social media campaigns was found, *differential* mobilization effects were evident when specific subgroups of the population were more closely examined (19).

Previous research in the marketing field, indeed, has consistently shown that targeted campaigns are much more effective than broad-reach campaigns in producing intended behavioral effects (20, 21). Randomized controlled trials in marketing report that microtargeting produces 150 to 183 % more profits than conventional advertising (22). Microtargeting increases desired behavioral effects by 135% in conservation and restoration behavior among landowners (23). Microtargeted advertising was 66% more effective than nonmicrotargeted advertising in social marketing (24). Moreover, recent experiments on issue advocacy campaigns also indicate that issue-specific microtargeting produces 70% larger persuasive effects on issue attitudes (25), suggesting a substantial impact of targeted political campaigns.

Although not specifically concerning targeted digital voter suppression, a few prior studies empirically assessed the IRA’s influence on social media (more specifically, X, formerly Twitter). For instance, Ruck et al. found that approximately every 25,000 additional IRA retweets predicted a one percent increase in election polls for Trump (26). Other studies found conflicting results. Based on a 2017 postelection partisan panel survey, Bail and colleagues indicated little evidence that the interaction with the Russian troll accounts changed political attitudes (27). Eady and colleagues’ simulation found that the following of the IRA was heavily concentrated among Republicans, yet no evidence of changes in their self-reported vote choice (28). However, none of those studies measured an individual’s *direct* exposure to an IRA message. Rather, they *presumed* exposure based on proxy indicators of potential indirect exposure (e.g. “Following”). As Aral and Eckle point out, however, no adequate causation is possible when no measure of direct exposure exists (29). Furthermore, because those proxies are often the outcome features of users’ own self-selections, it may even confound presumed effect estimations.

The present study strives to fill the void. First, by employing a user-based, real-time ad exposure tracking app, the study directly measures each individual’s exposure to targeted ads and captures ad content with metadata. Direct ad exposure measures enable us to measure exposure to a particular ad precisely, allowing examination of targeting patterns and potential effects that may suggest causal pathways. By merging with the survey responses of the individuals exposed to the ads, we examine the targeting patterns of voter suppression ads. By matching the individual exposure to voter suppression ads with the same individual’s voter turnout record, we estimate the effects of voter suppression ads on turnout while controlling for covariates. Second, when we identify voter suppression, we only examine ads (paid promotions), not unpaid messages (organic posts). Unlike unpaid posts, all Facebook ads—and digital ads in general—appear to targeted individuals only by design. An individual is exposed to an ad because she is targeted, not because she chooses to be. Ad exposure, thus, is unconfounded with users’ self-selection, which adds validity to causal inferences. Third, we track and match individuals’ *actual voter turnout records* with the same individuals’ voter suppression ad exposure before (no)voting, thereby establishing a clear time order for causal inference. Previous studies (27) estimated turnout based on self-reported willingness to vote or self-reported vote choices. Self-reported measures, however, suffer from a low level of validity, including overreporting of voting (30, 31). Fourth, the present study focuses on heterogeneity—heterogeneity in exposure (i.e., differential patterns in targeting by subgroups) and heterogeneity in effects (i.e., differential effects between subgroups). While randomized controlled trials have an advantage for precise causal estimates of the average effects on the population, it has a limitation in understanding the real-world effects, especially when the heterogeneity of the “treatment” (in this case, voter suppression exposure) is expected to be high (32). Given the reports that voter suppression efforts were narrowly targeted (3–5), a heterogeneous treatment effect (HTE) analysis based on observational data would be a necessary complement. Our relatively large sample size allows us to examine HTEs.

The lack of prior research of this kind is partly due to methodological challenges in studying digital ads on social media. By nature, digital political ads are shown to targeted individuals only. Even if select researchers obtain digital ad data through platforms, such observational data are often only available at the aggregate level, and it is impossible to identify and measure individuals’ ad exposure. Such data have fundamental limitations in characterizing targeted individuals or assessing the effects of individuals’ ad exposure on their electoral behavior. To address these challenges, we employ unique methodological approaches.

## User-Based, Real-Time Ad Exposure Tracking and User Turnout Tracking

We developed a user-based, real-time, digital ad exposure tracking tool (EScope) that works like an ad-blocker. Instead of blocking the ads, the app automatically detects, captures, and transfers ads and associated meta-information—such as landing pages, timestamps, and the individual identifiers of the app users exposed to the ads—to the research server. The tool enabled us not only to capture all the ad content but also to track the sponsors/sources behind political campaigns, even if the sponsors were not clearly indicated in ads. Furthermore, because this is a user-based tracking tool, we were able to identify each individual user’s ad exposure (33). The participants were asked to use EScope for approximately six weeks prior to the 2016 US general elections, from September 28 to November 8, 2016 (Election Day), which aligned with the FEC window, i.e., the campaign disclosure requirement period. The participants of the study were recruited by a firm, *GfK* (formerly Knowledge Panel), which specialized in online participant recruitment and sampling. Approximately 13,500 individuals who had given their consent used EScope. Overall, the sample represented the US voting-age population (VAP) in terms of gender, race/ethnicity, education, household income, age, state/region, and voter registration status. We did not detect any statistical differences between EScope adopters and nonadopters; the study participants and the VAP in terms of the aforementioned variables (*SI Appendix*, Table S1).

In conjunction with the ad data collection, we also administered EScope user surveys, which included demographics, geographics, and political profiles (e.g., party ID). By matching the survey responses of each EScope user to the ads they were exposed to, we examined the characteristics of users who were exposed to particular ads, thereby identifying targeting patterns and profiling targeted voters. A total of 10,441 consented individuals completed the baseline survey.

After the 2016 presidential election, we tracked the participants’ *actual* turnout records by using the voter files from each state. Matching between the study participants’ unique anonymized user ID, personally identifiable information (PII), and voter file was conducted by a collaborative team between the firm (GfK) who recruited our participants from their permanent study participant pool and assigned the individual’s user ID that the research team also used for the study and another firm (TargetSmart) that specialized in voter file aggregation and matching. Only the matched outcomes without PII were returned to the research team; thus, the research team handled no PII. We consider matched cases when all the PII were *exactly* matching. By exactly matching ad data and actual turnout records at the individual level, we were able to estimate the effects of ads on individuals’ turnout in the elections. For an overview of the study procedure, see *Materials and Methods*. For methodological details and robustness checks, see *SI Appendix*.

## Voter Suppression Ads

Following previous research (7), we define voter suppression as a strategy that demobilizes, discourages, or prevents specific segments of the population from voting. By nature, voter suppression is a strategy devised to break the coalition of the opposition and decrease the turnout of voters who are likely to support the opposition (7). Given this, we identify voter suppression ads based on targets’ party identity (target attribute) as well as ad content (message attribute).

We developed a dictionary that includes the terms frequently used in voter suppression ads based on seed data, where we observed Russian election interference (4, 11). We identified four types of voter suppression ads: election boycott, deception, third-party candidate promotion, and same-side candidate attack (see *SI Appendix* for details). For the primary analyses presented here, we used all voter suppression types, a total of 59,771 voter suppression ads. The accuracy rate—the proportion of accurately identified voter suppression ads (34)—was 0.93. All of them were sponsored by undisclosed campaigns, i.e., campaigns that did not report campaign activities to the FEC or the IRS (For details on the identification of voter suppression ads, including accuracy rate calculations, see *SI Appendix*, Methods 3.1).

## Targeting Patterns: Geo-Racial Targeting

Once voter suppression ads were identified, we first examined targeting patterns of voter suppression. While voter suppression is untethered to one particular political party (7), prior research (9, 35) indicates that historically, voter suppression has been heavily used to demobilize racially marginalized groups—African Americans in particular. Voter suppression is also strongly linked to some geographical attributes. Studies have found that voter suppression increases in competitive election environments (36, 37), including competitive districts and battleground states. As voter suppression targets specific types of voters, geographical attributes can be used to identify and target such voters as a proxy. Non-White voters, for instance, tend to be geographically concentrated; thus, counties with a high density of racial minority populations serve as a good proxy for targeting non-White voters (38). Indeed, racial gerrymandering is a strategic, geopolitical device that is designed to suppress the turnout of racial minorities and to prevent them from electing their preferred candidates. A body of research on voting rights and disenfranchisement of African American voters, for this reason, studied minority communities intensively (36, 39, 40).

Drawing upon the previous studies, we investigate whether and how digital voter suppression ads were disproportionately concentrated among non-Whites, especially non-Whites who reside in “minority counties,” where the proportion of racial/ethnic minorities is over 50% of the county’s voting age population per conventional definition in electoral studies (41, 42). We also examined “battlegrounds,” where the state’s vote margin was less than 5% in the 2016 presidential election, following prior research on battleground states (43).

**Fig. 2.**
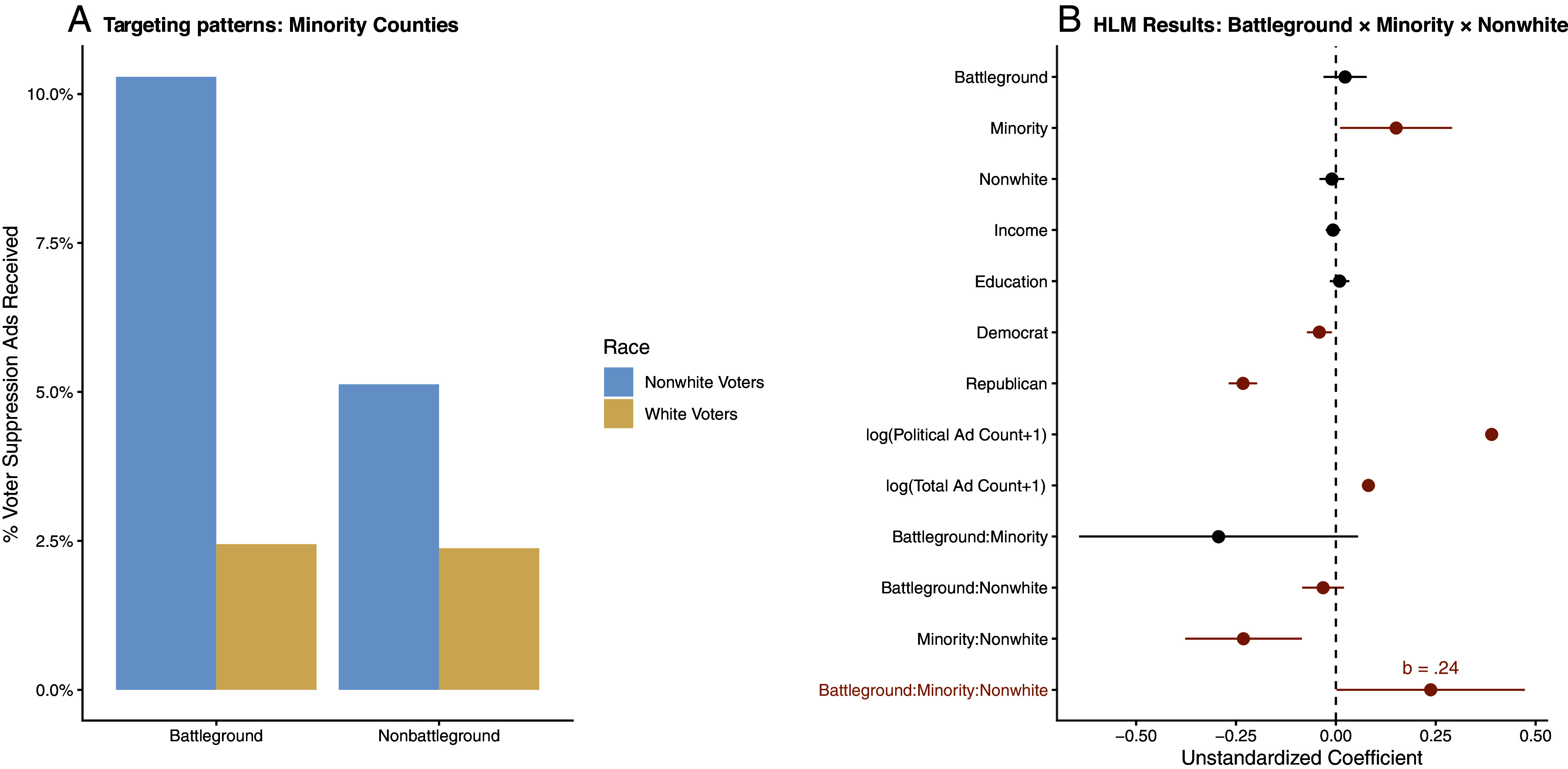
Targeting patterns. (*A*) Non-White voters living in minority counties in battleground states received disproportionately more voter suppression ads. For alternative figures, *SI Appendix*, Supplemental Analysis 1. (*B*) Plots indicate unstandardized coefficients and 95% CI obtained from an HLM where the interaction between non-Whites, battlegrounds, and minority-majority counties was tested after controlling for income, education, and party ID. Orange-colored dots and error bars indicate statistically significant coefficients with SE. 2. (battleground: minority; non-Whites) b = 0.24, *P* = 0.04 N = 11,943. Groups: 1,987. AIC = 36050.9.7, Log Likelihood = −18,004.4, Var: groups (intercept) = 0.034, var. Battleground = 0.175, var. Minority-majority counties = 0.086.

Fig. 2*A* shows the percentage of the voter suppression ads received in minority counties (see formula in *SI Appendix*, Methods 4.1). It indicates that non-White voters, in general, received more voter suppression ads than non-Whites. Non-Whites in battlegrounds received substantially more voter suppression ads than non-Whites in nonbattlegrounds, making the differential geo-racial targeting even more substantial.

Some scholars might argue that racial voter suppression occurs simply because non-Whites tend to be Democratic in the current political environment (44). In addition, targeting non-Whites (African Americans, in particular) with voter suppression efforts may also be reflective of the fact that they tend to have lower socioeconomic status (45). To rule out such possibilities, and to adequately investigate the interactions between the individual- and contextual-level attributes, we employed a Hierarchical Linear Model (HLM) (46), with the number of voter suppression ads an individual received being the dependent variable (*SI Appendix*, Methods 4.1.

Fig. 2*B* indicates that even after controlling for the individual’s household income and educational attainment, the geo-racial targeting patterns remain the same: non-Whites living in the minority counties in battlegrounds received significantly more voter suppression ads than their counterparts.

## Exposure to Voter Suppression Ads Is Associated with Decreased Turnout—But Effect Size is Small

We examined whether and how individuals’ prior exposure to voter suppression ads was associated with voter turnout. We tracked each of our participants’ *actual* turnout records in the 2016 presidential election (*SI Appendix*, Methods 2.4). The distributions of voter turnout rates observed in our sample (with missing imputation, PU-Learning; *SI Appendix*, Methods 3.5 and Technical Notes 1) closely resembled those of the population (65%) as well as relevant subpopulations (i.e., non-Whites, minority counties, and battleground states), lending support to the turnout model and the representativeness of the sample (*SI Appendix*, Table S3). Note that voter turnout records indicate actual voting, which occurred *after* the participants were exposed to the ads.

Considering the exposure to a voter suppression ad as a treatment, we identified those who were exposed to voter suppression ads (Exposure group: treatment group) and those who were not (Nonexposure group: control group). We first identified exactly matched pairs where all the values of the 35 predetermined covariates were identical [i.e., exact matching (47)]. Everything else being equal, the average voter turnout rate of the exposure group was *lower* than that of the nonexposure group by 5.7%.

Exact matching is the most stringent and conservative method in causal inference (47, 48). However, since it eliminates all the unmatched cases when comparing the treatment and control groups, it often results in an insufficient sample size for statistical power.

For more adequate, generalizable statistical estimates, we employed entropy balancing (49) as the identification and adjustment strategy for causal inference. As described in Hainmueller (49), it is a preanalysis weighting method for causal inferences based on observational data with a binary treatment. It achieves covariate balance through reweighting and assigns entropy balance weights to the control group. Thus, entropy balancing ensures that the treatment (exposure) and control (nonexposure) groups are identical in terms of the distributions of covariates (in our case, 35 covariates). This covariate adjustment thereby allows inference that any remaining difference between the two groups in voter turnout rates after entropy balancing can be attributed to exposure to voter suppression ads (49) (*SI Appendix*, Methods 4.2 and Technical Notes 2).

**Fig. 3.**
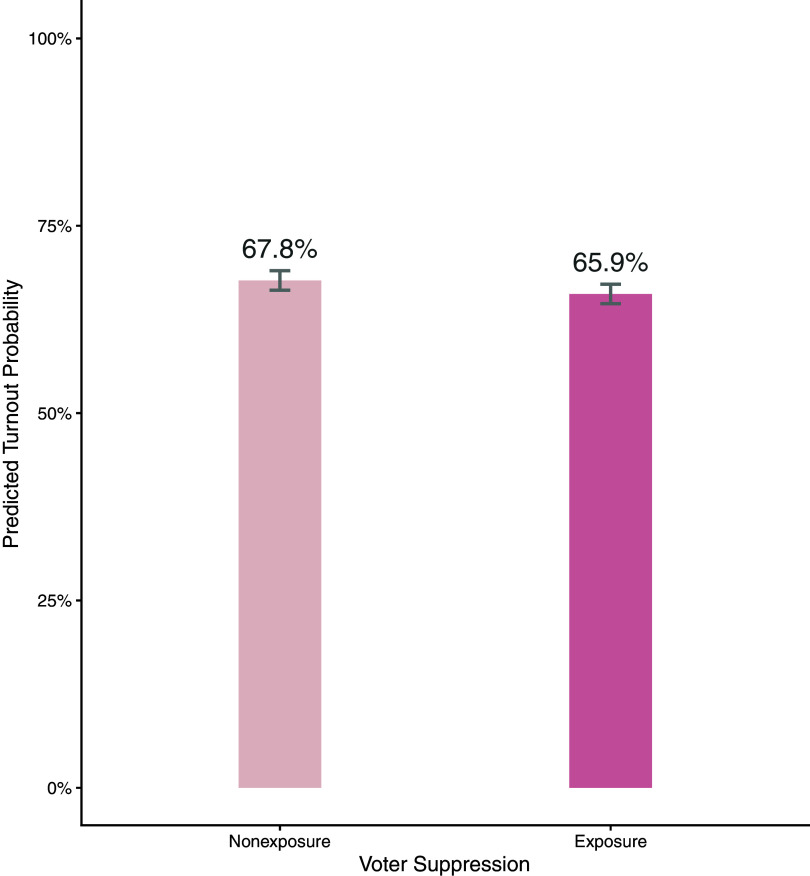
ATE of voter suppression on voter turnout. The effect of exposure to voter suppression on the individual’s turnout to vote. The numeric values on top of the bars indicate estimated group means after entropy balancing weights were applied. For the full-scale distributions, see *SI Appendix*. *b* = −0.019, *t*(8,546) = −2.72, *P* = 0.006. Cohen’s *d* = 0.059.

Fig. 3 shows the estimated ATE: the voter turnout rate of the exposure group was significantly lower than that of the nonexposure group, even after balancing covariates. Given that exposure to voter suppression was the sole difference between the two groups, and it clearly preceded actual voter turnout in our data, the findings suggest that exposure to voter suppression ads decreases the likelihood of voter turnout. On average, those exposed to voter suppression ads were less likely to turn out to vote by 1.86% (nonexposure: 67.75% vs. exposure: 65.89%), which amounts to approximately 4.7 million fewer votes nationally. To understand the magnitude of voter suppression effects in the context of the 2016 presidential election, it is worth noting that the vote margin between Clinton and Trump was 2%, equivalent to 4.9 million votes. Trump won by less than 1% of a vote margin in some battleground states, such as Michigan, Pennsylvania, and Wisconsin (Michigan 0.27%; Pennsylvania 0.72%; Wisconsin 0.77%).

One might argue that the estimated effects might have been driven by unobserved or unmeasured variables that we did not account for in covariate adjustments. To address this concern, we conducted sensitivity analyses with 35 covariates, including race (Whites vs. non-Whites), income, education, political ideology, party ID, policy issue importance, candidate preferences, voter turnout history, and so forth (50). The results of the sensitivity analysis indicated that the observed differences between the exposure and nonexposure groups in terms of turnout probability were unlikely to be explained by potential confounders or unobserved variables (*SI Appendix*, Methods 5.2, especially see *SI Appendix*, Fig. S5).

The effect size (Cohen’s *d*), however, was small (Cohen’s *d* = 0.059). To examine the robustness of the results, we also replicated the analyses using various types of control (counterfactual) groups (*SI Appendix*, Fig. S3 and Methods 5.1.1). We compared the turnout of those who were exposed to a voter suppression ad (treatment) to a) those who were not exposed to any voter suppression ads (no voter suppression exposure, the control group in this analysis; Counterfactual-A in *SI Appendix*, Fig. S3); b) those who were not exposed to voter suppression ads, but exposed to other political ads (no voter suppression political ad exposure; control group-b or Counterfactual-B in *SI Appendix*, Fig. S3); or c) those who were not exposed to any political ads (no political ad exposure; control group-c or Counterfactual-C in *SI Appendix*, Fig. S3). We used the same entropy balancing strategies, with the treatment being exposure to voter suppression.

While there was a modest level of association between those who were not exposed to voter suppression and those who were not exposed to any political ads, we found that voter suppression exposure was distinct from exposure to other political ads, both in terms of its targeting and effects. Specifically, we did not observe geo-racial targeting patterns in other types of political ads. Additionally, those with no exposure to political ads did not share similar individual- or contextual-level characteristics with those exposed to voter suppression ads. Furthermore, even compared to those not exposed to voter suppression ads but exposed to other political ads, those exposed to voter suppression exhibited a lower level of turnout (control group-b), although statistically insignificant. In contrast, no significant difference in turnout was observed between individuals exposed to voter suppression ads and those who received no political ads at all (control group-c). Given the findings, we further examined the associations between nonvoter suppression political ad exposure (treatment 2) and voter turnout. If it generates mobilizing effects, it indicates the distinctive effects of voter suppression ads. As expected, exposure to political ads that were not intended to suppress voter turnout was associated with an increase in turnout.

We further performed additional robustness checks (for details, *SI Appendix*, Methods 5)—a false-shock test (*SI Appendix*, Methods 5.3.1), a placebo test with the 2012 turnout records (*SI Appendix*, Methods 5.3.2), and alternative effect estimates using different identification and adjustment techniques (*SI Appendix*, Methods 5.4). The results withstood all tests, confirming the robustness of our findings.

## Voter Turnout Is Lowest Among Geo-Racially Targeted Segments

While the population average treatment effect (PATE or ATE) is a key finding of this study, it does not fully capture the nuanced effects of targeted voter suppression, as we controlled for both individual-level (race) and contextual-level (minority counties, battlegrounds) geo-racial targeting attributes in the identification and balancing. In other words, the ATE illustrated in this study captures the nondiscriminant message effects of the voter suppression ads across different segments of the population.

Given the geo-racial targeting patterns observed in voter suppression ads (Fig. 2), we would have a better understanding of the effects of geo-racially targeted voter suppression ads by examining the HTE of race (individual-level targeting attribute) and geographical targeting attributes (minority counties and battleground states as contextual-level targeting attributes). Given that our targeting pattern analysis indicated that voter suppression ads specifically targeted non-White voters in minority counties of battleground states, what matters the most is the heterogeneous effects of the ads on those targeted segments of the population rather than the population average effects of a voter suppression ad message. Thus, our HTE tests focused on the differential effects of voter suppression ads on the targeted segments of the population we empirically detected in our targeting pattern analysis (Fig. 2): non-Whites, those residing in minority counties, and those in battleground states. We observed voter turnout among different geo-racial segments of the population (for the descriptive statistics of the groups, see *SI Appendix*, Table S4). Modeling HTEs with interactions between voter suppression exposure and race, state, and county, we applied entropy balancing reweights based on split samples, then estimated differential effects across subgroups (*SI Appendix*, Tables S5 *A* and *B* and Supplementary Analysis 2).

**Fig. 4.**
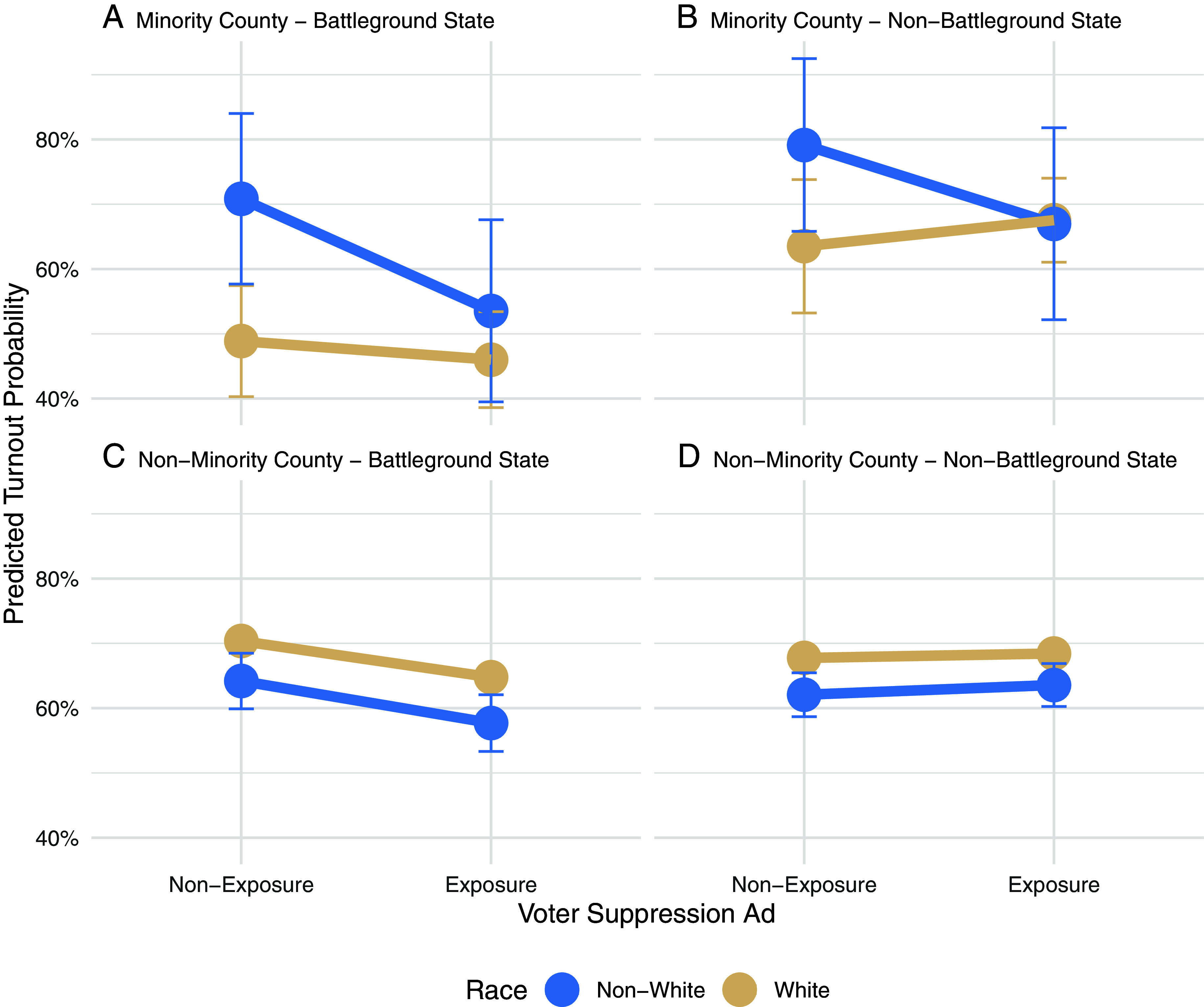
HTE (voter suppression exposure × race × minority county × battleground state). Estimated voter turnout between voter suppression exposure vs. nonexposure and non-Whites and Whites in (*A*), Residents of the minority counties within battleground states; (*B*), minority counties in nonbattleground states; (*C*) nonminority counties in battleground states; and (*D*) nonminority county in nonbattleground states. This figure is based on the four-way interaction model where exposure (treatment) and nonexposure are balanced with entropy balancing, while the treatment is fully interacted with race, county, and battleground variables. Circles indicate the unstandardized estimates, and lines describe the 95% CI based on a linear regression model where voter suppression exposure (exposure vs. nonexposure), race (non-Whites vs. Whites), counties (minority vs. nonminority), states (battlegrounds vs. nonbattlegrounds), full interaction terms are included. *F* (15, 8,532) = 7.63, *P* < 0.001, R-square = 0.013, RSE = 0.227. Large CI of some subgroups indicate low precision, although point estimates are robust. Alternative split sample analysis results indicate relatively high precision and robust estimates (*SI Appendix*, Supplemental Analysis 2 and Fig. S7), while generating consistent patterns observed here. Voter turnout rate in minority counties was low in general, perhaps due to the “(minority) neighborhood effect”—contextual effects of residing in a racially minority group dominant county. The voter turnout of the minority counties in battlegrounds was already significantly lower compared to other geographical areas. In minority counties, White voters’ turnout rate was lower than that of non-Whites in the control group (no voter suppression exposure), possibly because of the community-level turnout mobilization efforts (e.g., GOTV) for non-Whites when no individual-level racial targeting was available (*SI Appendix*, Supplementary Analysis 4 for further discussion).

As shown in Fig. 4, while turnout depression was substantially amplified among non-White voters, voters in battleground states, and those living in minority counties, voter turnout rates were lower especially among voters in battlegrounds (−5.8%, Cohen’s *d* = −0.185), non-White voters in battlegrounds (−7.5%, Cohen’s *d* = −0.221) or non-White voters in minority counties (−15%, Cohen’s *d* = −0.520). The voter turnout probability was the lowest among non-Whites in minority counties of battleground states. The treatment effect size of non-Whites in minority counties of battleground states was relatively large (−17.3%, Cohen’s *d* = −0.515). Even after accounting for variability across and within different segments of the sample in terms of race, counties, and states, the association between exposure to voter suppression and decreased voter turnout rates was pronounced. When non-White voters of minority counties in battleground states were exposed to voter suppression ads, their turnout rate was 14.2% lower when compared to the complete counterpart segment, White voters in nonminority counties of nonbattleground states who were not exposed to voter suppression ads (Cohen’s *d* = −0.450). It appears that the differences in voter turnout after exposure to voter suppression ads were largest among geo-racially targeted segments, especially non-Whites in battlegrounds. To contextualize, the voter turnout difference between the White and non-White subpopulations in the 2016 Presidential Election was about 12%, according to the Census data (*SI Appendix*, Table S3).

Altogether, the findings suggest that the significant differences in voter turnout rates among geo-racially different segments of the population, in part, can be explained by geo-racially targeted voter suppression ads.

As another robustness check, we sought to isolate the effect of the exposure to targeted voter suppression ads as best as possible by comparing the turnout of the targeted segments—in this case, non-Whites in minority counties of battlegrounds—who were exposed to voter suppressions and that of the same targeted segments who were not exposed to any voter suppression ads (control group-d, or missed exposure group; “nonexposure” among targeted segment in *SI Appendix*, Fig. S4*A*). This test is designed to provide a better understanding of the robustness of the observed differences in voter turnout rates across and within subgroups. For comparison, we also compare the turnout of the targeted exposed group to those who were exposed to voter suppression ads but do not fit the targeted segment, specifically White voters in nonminority counties of nonbattleground states (control group-e, mistargeted group; “mistargeted” in *SI Appendix*, Fig. S4*B*). This test would provide insight into how strong geo-racial targeting would be even among those who were exposed to voter suppression ads.

Given that targeting is a necessary and sufficient condition for ad exposure on social media—ads are delivered only to targeted individuals—true counterfactuals would not exist in reality if geo-racial targeting were executed with perfect precision. However, these comparisons enable us to address whether the observed differences were primarily driven by the individual or contextual attributes of the targeted segments (geo-racial targeting itself) rather than the actual exposure to targeted voter suppression ads.

If the observed patterns still persist even when compared to different counterfactuals, this strengthens the inference that exposure to targeted voter suppression ads explains reduced turnout. Especially if the decrease in voter turnout rates is larger when compared to the missed exposure group (control group-d, or missed exposure group: *SI Appendix*, Fig. S4*A*) than when compared to the mistargeted group (control group-e, mistargeted group: *SI Appendix*, Fig. S4*B*), it suggests that the voter suppression are not just driven by the individual and contextual characteristics of the targeted segment, but much explained by the exposure to targeted voter suppression ads and its interaction with contextual and individual attributes.

The results (*SI Appendix*, Fig. S4) show that the turnout rate was significantly lower—indeed, the lowest—among the targeted exposed group compared to the missed exposed group or the mistargeted group, suggesting the robust heterogeneous effects of geo-racially targeted voter suppression ad exposure on voter turnout.

## Discussion

Independent of governments or social media companies, we document the patterns and impacts of targeted digital voter suppression advertising by undisclosed groups, including the Russian IRA. We empirically examined its targeting patterns and estimated its effects on turnout by tracking the digital ad exposure of a large number of voting-eligible individuals for approximately six weeks leading up to the 2016 election day and later matching voter suppression exposure with their surveys, as well as actual voter turnout records at the individual level. In light of recent discussions about the challenges in social media research—including those arising from academic-industry collaborations, particularly researchers’ limited data access and control over research protocols (51)—our independent data collection and research design provide a complementary perspective.

Several findings are worth highlighting. First, the results of this study show that even after controlling for individual or contextual attributes, non-White voters in minority counties of battleground states were exposed to voter suppression ads significantly more than their counterparts, indicating geo-racial targeting of voter suppression campaigns. The results corroborate US intelligence reports that Russian election interference, in part, targeted non-White voters with voter suppression ads, but also echo other reports regarding the infringement of voting rights on social media.

Second, on average across the population, exposure to voter suppression ads was associated with a 1.9% decrease in voter turnout. The sensitivity analysis indicated that the estimated effects were robust and unlikely to be explained by unobserved confounders in a plausible magnitude. We conducted a series of robustness checks, including tests with different control groups, analyses with various identification and adjustment techniques, and placebo tests; the results were robust and consistent. Given that the popular vote margin between Clinton and Trump was about 2% and that the winner was determined by less than a 1% margin in many states, the 1.9% decrease in voter turnout is indeed notable. However, the effect size is marginal, warranting caution in interpretation.

Third, the voter suppression effect greatly varied by different subgroups, indicating significant HTE—the estimated effects were large among targeted segments of the voters, non-Whites in minority counties of battleground states. The voter turnout difference between this targeted segment and its complete counterpart was 14.2%, with a large effect size. The effects remain robust even when tested with different control groups. The findings lend support to previous research indicating that targeted campaigns may be more effective than those randomly distributed to the general public.

At first, our findings might appear inconsistent with those of the previous studies that made causal inferences with observational data, which evidenced a null effect of the IRA (27, 28). However, differences in research designs, measures, and analytical strategies must be noted. First and foremost, this study investigated targeted ads (Facebook), while previous studies examined nonpaid, nontargeted messages (more specifically, tweets). Second, previous studies did not directly measure exposure. Their “exposure” measures, in fact, assumed exposure based on the interactions or relationships with Russian trolls, such as following accounts, tweets, or retweets. By contrast, this study directly measured each individual’s first and direct exposure to the ads that targeted the very same individual. Given that individuals are exposed to ads on the platform only if they are targeted, our direct exposure is free from self-selection. Third, while the outcome variables of the previous studies were measured by self-reported surveys on issue positions, candidate preferences, and the like, the outcome of this study is the study participants’ actual voter turnout records that were exactly matched at the individual level. Given that self-reports, especially self-reported turnout, tend to be highly conflated, actual turnout records observed in this study would have higher measurement validity.

Furthermore, given that voter turnout occurred clearly after the voter suppression campaigns we observed in this study, and it was impossible for ad sponsors to know exactly when it would happen at the time of individual ad exposure, this study addresses a potential endogeneity concern through a clear time order. It is also important to note that while this study investigates undisclosed campaigns, including Russian election interference, it focuses on a specific type of message: voter suppression. On the other hand, previous studies examined Russian troll accounts altogether without specifying the various types of messages users interacted with. Given that Russian election interference targeted both sides of the ideological spectrum with customized messages designed to appeal to each side independently yet simultaneously (3), we might miss nuanced differences in estimating effects if the type of message is not specified.

Of utmost importance is that, in the data-driven, microtargeted, digitally mediated campaign environment, even the same campaign sponsor (e.g., the IRA) could target different segments of the population with different messages; hence, the effect of campaigns would widely vary by differences in targets and messages even within the population. Especially when a particular type of message is strategically designed to target a narrow segment of the population with distinct characteristics, it might be unreasonable to expect the invariably broad impact on the general population presumed in the broadcast media era, when everyone was exposed to the same message with a random chance of exposure. As found in this study, even when we specifically focus on vote suppression, the average population effect size was relatively small. In contrast, the differences were pronounced among the targeted segments within the population. When the previous studies heavily focused on the average population effect (ATE) of overall troll accounts, a null effect might not be surprising. With such an approach, however, we might inadvertently neglect even the intended effects of strategically narrow targeting and messaging based on an unsustainable assumption of the invariable broad impact that was only possible in the broadcast era. Future research must consider the evolving nature of the information environment and pay closer attention to more nuanced heterogeneous effects on target audiences of interest within the general population at both the theoretical and methodological levels.

The results of this study shed light on our understanding of targeted ads in the modern-era data-driven, digitally mediated information environment and its implications for the functioning of democracy. The findings of the study suggest that geo-racially targeted voter suppression messages, especially when targeting marginalized voters, effectively lower actual—rather than stated—voter turnout. This raises a normative concern for democracy, as it indicates an unequal distribution of representation in the most fundamental form of democratic participation. It is even more deeply troubling that such voter suppression ads were sponsored by undisclosed groups—including the ones that later turned out to be the IRA, a disinformation campaign operation supported by the Kremlin.

Despite potential contributions, this study has limitations. While the results remain consistent and withstand a series of robustness tests, and while the findings corroborate previous intelligence reports on targeted ads and the Census data on turnout patterns—suggesting both high internal and external validity—causal inference nevertheless requires caution. Even though the sensitivity analysis suggests that the findings are robust to plausible unobserved or unmeasured confounders, and the placebo tests further provide little evidence for spurious effects, causal inference based on observational data is inherently limited. Although observational data better reflect reality and hence have merit in broad generalization, this study draws exclusively on specific cases from the 2016 US Presidential Election. Accordingly, the interpretations should be made with caution, given the specific context. Future research should further explore such inquiries in other election contexts with various other research designs and analytical techniques to build a more comprehensive understanding of targeted digital voter suppression and its impact on voter turnout.

## Materials and Methods

The present study is part of a larger research project, Project DATA (Digital Ad Tracking & Analysis), that tracks digital advertising across multiple platforms, its sponsors/origins, content, targets, and effects by employing user-based, real-time, ad exposure tracking and associated user surveys, geographics, and individual-level voter turnout records. This study focuses on voter suppression ads on Facebook during the 2016 US Presidential Election, where voter suppression by groups originating in Russia on social media was extensively discussed, yet its effects on actual voter turnout were, to the best of our knowledge, not empirically investigated.

**Fig. 5.**
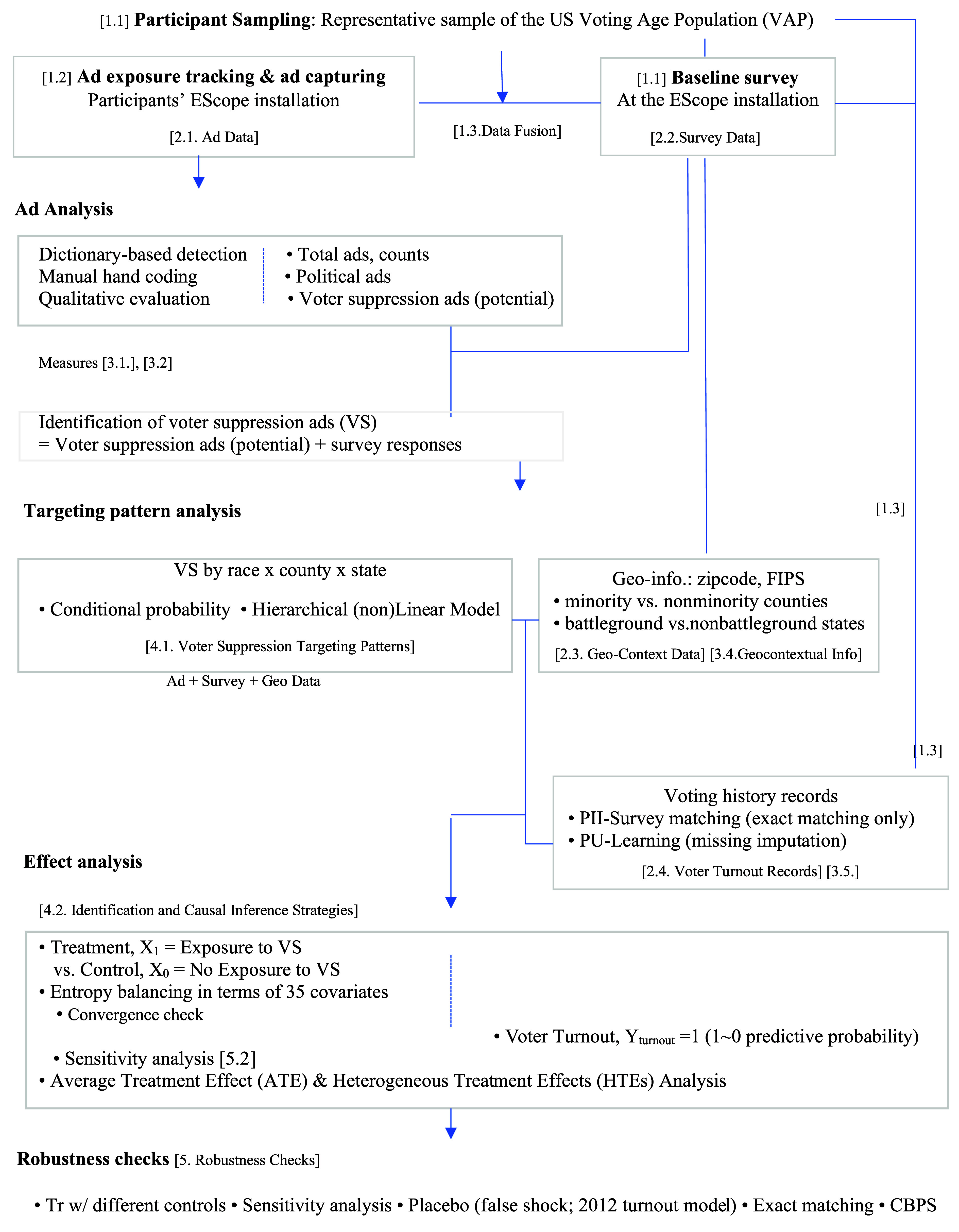
Overview of methods.

Fig. 5 illustrates an overview of methodological procedures: participant recruitment and sampling; digital ad exposure; offline behavior tracking; four datasets (ads, survey, geo-context, voter file data); data fusion (how data are combined at the individual level); measures; analytical techniques; and robustness checks. For each stage, Fig. 5 includes the corresponding numbers of the SI sections where detailed descriptions are provided.

As shown in Fig. 5, this study employed a sample of the US voting-age population recruited by a sampling specialty firm, GfK. Participants provided informed consent and were asked to use a user-based, real-time ad tracking tool, EScope, that directly measured each user’s ad exposure, while capturing ads with associated metadata. Participants used the app for about six weeks leading up to the election day of the 2016 Elections. At the installation of the app, participants were also asked to complete a baseline survey that included a questionnaire on demographics, political party identity, voter registration, perceived issue importance, candidate preferences, and so forth. Because the participants were permanent panelists of GfK, their personally identifiable information (PII), such as names, birthdates, and home addresses, was recorded. We adopted the same anonymized user IDs GfK permanently assigned to each participant when tracking ad exposure and implementing surveys. We partnered with GfK and a third-party firm (TargetSmart) specializing in collecting individuals’ voter turnout records; thus, our team was able to have GfK and TargetSmart work together on our behalf to track and match our participants’ voter files after the study was completed. GfK then sent back each participant’s voting history records with the anonymized user ID, stripping away PII. In this way, we successfully tracked and exactly matched voter files at the individual level, even though we did not collect our participants’ PII. When matching our participants’ data with voter files, we only used cases where *all* the personally identifiable information (PII) *exactly* matched that of the voter files. By nature, voter profile data are “presence-only” data—it contains observed positive cases only (Y_turnout_ = 1) with the “unlabeled” in matched cases, or “unmatched” cases, where the individual’s actual voter turnout status is unknown. In this case, it is important to discern missing cases from true no turnout (Y_turnout_ = 0) and true turnout (Y _turnout_ = 1). As a technique for missing value imputation, we employed the *PU-learning technique*, which accounts for such data structures (52). The sample’s turnout mirrored the actual turnout in the 2016 presidential election.

We first identified four types of voter suppression ads by employing a dictionary approach and human coders’ validity check processes. The intercoder reliability (Krippendorff’s alpha) (34) was 0.93 (election boycott 0.91; deception 0.98; third party candidate promotion 0.94; candidate attack 0.92). By tracking the users exposed to a particular ad and merging ad and user-associated information, such as the user’s survey responses and associated geo-context data (e.g., zipcode), we “reverse engineered” the targeting profiles of an ad. In this study, once we identified voter suppression ads, we examined targeting patterns (especially geo-racial targeting, if any) by employing hierarchical linear modeling with voter suppression ad exposure (number of exposures) and regressing on user demographics and geographics.

By combining ad exposure, survey responses, geographics, and voter turnout records at the individual level, and by identifying the voter suppression exposure and nonexposure groups (exposure = 1, nonexposure = 0), we grouped the sample into “treatment (exposure)” and “control (nonexposure)” groups. We balanced the covariates between the exposure and nonexposure groups with *entropy balancing. Entropy balancing* (49) assigns weights to the control group respondents such that the distribution of covariates between control and treated respondents would be the same. It is a preanalysis processing method for causal inferences based on observational data with a binary treatment and achieves covariate balance through reweighting. The estimator is consistent for the Population ATE for the Treated (PATT), thus doubly robust (53). Entropy balancing works with a large set of covariates while keeping all the data, whereas exact matching, propensity score matching or nearest neighbor matching discards unmatched cases. It also frees us from repetitive specification by directly including covariate balance in the estimation procedure. With no model dependence, no linearity assumption is required regarding how outcomes are related to the treatment. With entropy balancing, the only difference between the treatment and the control group is whether participants were exposed to voter suppression (treatment) or not (control). Having entropy-balance reweights applied, we estimated the ATE and HTE.

To check the robustness of the results, we conducted: a series of reanalyses of the treatment effects with different control groups (counterfactuals); a sensitivity analysis (50); multiple placebo tests (false shock test and prediction for the 2012 voter turnout); and estimations with various other identification and adjustment strategies (exact matching, full matching with Covariate Balancing Propensity Score, CBPS) (54). The patterns were consistent with those of the primary analysis reported in the Main Text.

## Diversity, Equity, Ethics, and Inclusion and Statements on the Protection of Human Subjects in Research and Data Privacy and Security

The research has been approved by the Institutional Review Board for Protection of Human Subjects in Research at the University of Wisconsin-Madison (2015–1573). Sampling was random; thus, no inclusion or exclusion of target demographics applied, other than the voting-eligible US population. Only consented individuals participated in the research. Neither the browser extension nor the survey collected personally identifiable information. We did not collect users’ personal profiles or friends’ networks. Data access is strictly limited to the IRB-trained researchers in the team only. The data collection app works in a secure, encrypted Web server, and the data are stored on a secure data server. We have a server architecture that separates ad data, meta-information, and survey data. When matching ad data, its metadata, and survey responses, anonymized user identifiers (i.e., 36-digit user ID tagged at the installation of the app) were utilized for data analysis. The publication of data analysis includes aggregate-level information only.

## Supplementary Material

Appendix 01 (PDF)

## Data Availability

Research protocols are presented in *Materials and Methods*, *SI Appendix*, including *Technical Notes*. Data, materials, and codes for replication are available through OpenICPSR of the Inter-university Consortium for Political and Social Research (ICPSR) at the University of Michigan (https://doi.org/10.3886/E242583V1) (55).
