# Supplementary material for: Targeted digital voter suppression efforts likely decrease voter turnout
Source: Proc Natl Acad Sci U S A. 2026 Jan 26;123(5):e2519944123. doi: 10.1073/pnas.2519944123 (PMC12867748; doi:10.1073/pnas.2519944123)
Supplement: Supplementary file 1 — Appendix 01 (PDF) [file pnas.2519944123.sapp.pdf]

PNAS

Original Research Article

Running head: Targeted Voter Suppression

Supporting Information (SI)

TARGETED DIGITAL VOTER SUPPRESSION EFFORTS LIKELY DECREASE VOTER TURNOUT

Young Mie Kim[1]*, Ross Dahlke[2], Hyebin Song[3], Richard Heinrich[4]

[1] Vilas Distinguished Achievement Professor, the School of Journalism and Mass Communication and the Department of Political Science (Faculty Affiliate), University of Wisconsin-Madison; Madison, WI 53706, USA

[2] Assistant Professor, the School of Journalism and Mass Communication, University of Wisconsin-Madison; Madison, WI 53706, USA

[3] Assistant Professor, the Department of Statistics, Pennsylvania State University, University Park, PA 16802, USA

[4] Senior Consumer Insights Strategist, the Wisconsin School of Business, University of Wisconsin-Madison; Madison, WI 53706, USA

* Corresponding author. Email: ymkim5@wisc.edu

SUPPORTING INFORMATION (SI)

For an overview, see the Materials and Methods section of the Main Text

METHODS

1. Participant Sampling and Data Collection
   1.1. Participant Sample: Representative Sample of the US Voting Population
   1.2. Individual-Level Ad Exposure Tracking: User-Based, Real-Time Ad Tracking App
   1.3. Data Fusion: Merging with Ads, Surveys, Geographics, and Voter Turnout

2. Data
   2.1. Ad Data
   2.2. Survey Data
   2.3. Geo-Context Data
   2.4. Voter Turnout Records (Voter Files)

3. Measures
   3.1. Identifying Voter Suppression Ads
   3.2. Exposure to Voter Suppression Ads, Total Ad Exposure, and Total Political Ad Exposure
   3.3. Socio-Demographics, Political Attitudes, and Behavior Variables
   3.4. Geo-Contextual Information
   3.5. Voter Turnout: The Individuals' Turning Out to Vote

4. Analytical Techniques
   4.1. Voter Suppression Ad Targeting Patterns
   4.2. Voter Suppression Effects: Identification and Causal Inference Strategies

5. Robustness Checks
   5.1. Treatment Effects with Different Control Groups (Counterfactuals)
      5.1.1. Average Treatment Effects with Different Counterfactuals
      5.1.2. HTE with Counterfactuals: Conditional Treatment Effects (CTE) with Different Subgroups
   5.2. Sensitivity Analysis
   5.3. Placebo Analyses
      5.3.1. False Shock Test
      5.2.2. Prediction for the 2012 Voter Turnout
   5.4. Effect Analysis with Different Identification and Balancing Strategies
      5.4.1. Exact Matching
      5.4.2. Full Matching with Covariate Balancing Propensity Score (CBPS)

SUPPLEMENTAL ANALYSIS & ITS RESULTS

1. Unpacking Targeting Patterns
2. Unpacking Heterogeneous Treatment Effects
3. Effects of Different Voter Suppression Ad Types
4. General Mobilization (Get Out The Vote, GOTV) Effects
5. Voter Suppression Effects Conditioned on Timing

TECHNICAL NOTES: DETAILS OF ANALYSIS TECHNIQUES

1. PU Learning
2. Identification and Balancing Strategies and Causal Analysis Protocols

SURVEY QUESTIONNAIRE

Methods

1. Participant Sampling and Data Collection Strategies

1.1. Participant Sample: Representative Sample of the US Voting Population

The participants of the study were recruited by a firm, *GfK* (formerly Knowledge Panel), which specializes in online participant recruitment and sampling for social science research. GfK was the only company that did probability sampling for an online panel. Participants who consented to the study were asked to use EScope for approximately six weeks prior to the 2016 general elections, from September 28 to November 8, 2016. A total of 13,500 consenting individuals used Escope during the general election study period. The sample mirrored the US voting age population in terms of gender, race/ethnicity, education, household income, age, region (Northeast, Northwest, Southeast or Southwest) or state (50 states plus Washington D.C.), and whether they have registered to vote at the time of recruitment. For details on recruitment, sampling, and representativeness, see Kim et al. 2018. When compared to GfK pool of available participants, we also did not find any systematic biases between those who adopted EScope; those who filled out the baseline survey; and those who completed the baseline survey.

Table S1: Representativeness of the sample

Demographic Characteristic	Category	ACS	Sample (Weighted)	Sample (Un-weighted)	GFK
Income	Lower	37%	36%	35%	47%
Income	Middle	52%	53%	55%	47%
Income	Upper	11%	11%	10%	16%
Race	White	70%	70%	78%	78%
Race	Non-White	30%	30%	22%	22%
Age	18 to 24 years	13%	12%	15%	23%
Age	25 to 34 years	18%	16%	34%	
Age	35 to 44 years	17%	15%	23%	44%
Age	45 to 54 years	18%	25%	15%	
Age	55 to 64 years	16%	15%	10%	25%
Age	65+ years	19%	17%	4%	8%
Gender	Non-Female	48%	50%	35%	41%
Gender	Female	52%	50%	65%	59%
Education	No College Degree	65%	56%	55%	66%
Education	College Degree	36%	44%	45%	34%
Voter Registration	Not Registered	30%	5%	6%	20%
Voter Registration	Registered	70%	95%	94%	80%

ACS is the ACS 5-year, 2012-16, Voting Age Population (VAP). Race and gender are based on the Citizen Voting Age Population (CVAP). Voter registration in the ACS column is based on the Census's report on the 2016 Elections, whereas that of the sample is self-reported. Sample (Weighted) is benchmarked using the ACS 5-year, 2012-16. GfK indicates GfK's entire permanent participant pool.

1.2. Individual-Level Ad Exposure Tracking: User-Based, Real-Time Ad Tracking App

No social media ads, including Facebook ads, were accessible before Meta constructed the Ad Library in 2018; thus, no study ever had previously empirically examined digital (paid) campaigns on Facebook in the 2016 presidential elections. Even the current Ad Library only provides limited information about ad targets at the aggregate level (e.g., % of males vs. females targeted by an ad), lacking precise information about which individual is exposed to a specific ad.

By nature, a Facebook ad, which has the nickname of "dark post," only shows up to targeted individuals. Because no one other than a targeted individual is exposed to Facebook ads, observing and measuring individual-level ad exposure at scale is nearly impossible. The lack of an individual-level exposure measure makes it difficult to make causal inferences about the effects of targeted ads on the individual[1].

To overcome such challenges, we developed EScope, which works like an ad-blocker. However, instead of blocking them, the app automatically detects, captures, and transfers the ads (texts, images, texts on the images, videos, etc.) each user is exposed to, with the associated meta information—such as landing pages (where it directs users when clicked; often the ad sponsor's Pages or its external website), sponsor names and identities, the unique user identifier (anonymized individual-level identifier of the user who is exposed to the ad), and the timestamp of user exposure. This tool enabled our team to track the sponsors/sources of political campaigns, examine the content of the ads, and identify the specific users exposed to the ads with an anonymized unique individual user identifier (for details, see user-centric behavioral tracking[2]).

We checked whether any systematic biases existed between those who adopted Escope and those who did not by obtaining information about GfK's entire participant pool, as well as non-adopters. We did not find any systematic differences between the adopters and non-adopters.

1.3. Individual Attributes and Offline Electoral Behavior Tracking : Surveys, Geographics, and Voter Turnout Tracking

At the installation of the ad tracking tool, we asked participants to fill out a baseline survey. A total of 10,441 individuals completed the baseline survey (see Survey Data). We also collected each participant's zip code, which was later used to identify each participant's county-level residential area. By merging the ad data and associated user-level information, including survey responses and geographical information, we were able to "reverse-engineer" and model targeting profiles of each ad.

Furthermore, unlike most prior research employing self-reports on turnout—which is often overestimated[3]—we tracked our participants' actual turnout records in the 2016 elections by using voter files from each state. The matching between the study participants' unique anonymized user ID, personally identifiable information (PII), and voter file was conducted as a collaborative effort between GfK and TargetSmart, which specializes in voter file compilation and matching. Only exactly matched outcomes with anonymized unique individual identifiers were returned to our research team. The unique individual identifiers assigned to each individual user were the same as those we used for ad tracking and surveys. By matching ad data and actual voter turnout records at the individual level, we were able to track each participant's ad exposure and the same person's voter turnout record, along with individual-level attributes obtained from survey responses, thus estimating the effects of ads on individuals' turnout in the elections.

2. Data

2.1. Ad Data

The present study utilizes approximately 5 million Facebook ads exposed to about 13,500 study participants using EScope between September 28, 2016 and November 8, 2016. This includes any Sponsored News Feeds, feeds from Promoted Pages (both categories together about 1.3 million) and right-column ads (about 3.7 million) our study participants were exposed to during their normal online activities. We then identified political ads out of all the Facebook ads with a dictionary that contains frequently used terms in 32 sub-domains/topics in political ads (about 1.2 million, 23% of all ads). We also used the known political sponsor names in identifying political ads. Voter suppression ads were identified with a similar approach (see *Voter Suppression Ads* under **Measures**).

Because ad sponsors pre-determine targeting profiles that characterize their target audiences, it is crucial to note that individuals are exposed to digital ads *only because they are targeted*[4]. To reach out to these audiences, ad sponsors use various targeting methods provided by Facebook, such *as custom audience targeting*[5] (ad sponsors provide a list of specifically targeted individuals), *look-alike targeting*[6] (based on the custom audience target or target characteristics ad sponsors provided, Facebook finds the users have the same profiles), and *interest-based targeting*. In *interest-based targeting*[4,7], ad sponsors choose a specific issue and associated keywords, demographics, or geographics, (e.g., gun rights, “2nd Amendment”, “National Rifle Association, NRA”, “males 50 years or older, those living in Winnebago County, Oshkosh in Wisconsin”, et cetera), to narrowly target the individuals who are interested in the issue or topic. The Internet Research Agency (IRA), the Kremlin-linked disinformation operation, indeed employed interest-based targeting when engaging in election interference during the 2016 elections[8].

Regardless of Facebook’s targeting methods, an ad is only exposed to the individuals targeted by the ad sponsor, which is determined by the ad sponsor’s predetermined targets or target profiles[4]. Ad exposure is ***not*** determined by self-selection. This targeting and exposure mechanism makes paid promotions (Sponsored News Feeds, Promoted Pages, Right Column ads on Facebook) quite different from that of non-paid feeds (namely, “organic posts”), which are promoted by Facebook’s ranking algorithms. This implies that, unlike organic posts, Facebook ad exposure has relatively little to do with users’ self-selection biases or Facebook’s algorithms.

Given that targeting is a necessary and sufficient condition for exposure, by identifying and patterning the very individuals who are exposed to a specific type of ad, we can have a better understanding of target profiles. Once we captured voter suppression exposure at the individual level, we identified the individuals exposed to voter suppression ads and modeled voter suppression ad exposure by utilizing the individual-level attributes obtained from other data, such as survey and geo-contextual data. This “reverse engineering” technique helps us understand ad sponsors’ target profiles and targeting patterns and potentially renders more information than Meta itself, which does not necessarily have all the information on individual-level attributes of ad sponsors’ target audiences, such as race/ethnicity and party identity.

2.2. Survey Responses

A total of 10,441 users completed the initial baseline survey at the installation of EScope. The baseline survey included questionnaires on demographics (gender, race, age), socioeconomic status (education and income), party ID, and voter registration status. As shown in Table S1 in the previous section (2.1), the sample is generally similar to the US voting age population. However, to more closely represent the US voting age population, we weighted the sample based on the American Community Survey (ACS) 2012-2016 5-year estimates. Age, gender, and race were weighted such that the weighted distributions of the variables are identical to those in the US voting age population (cell weighting)[9].

2.3. Geo-Context Data

To examine geo-targeting patterns, we employed a couple of geographic variables (minority counties and battleground states at the state and county levels by matching each individual participant's residential area (zipcode, obtained by the recruitment firm, GfK) with external data such as the state-level vote margins and American Community Survey (the Census). For details, see *Battlegrounds*, *Racially Minority Communities (Minority Counties and Minority Congressional Districts)* under **Measures**.

2.4. Voter Turnout Records (Voter Files)

Most of the previous studies on voter turnout used inferred proxies (e.g., predicted values based on voter registration information) or self-reports, which tend to overestimate turnout modeling[3]. By contrast, we obtained actual voter turnout records of each of our study participants. By working with GfK, the firm that recruited and provided us with the sample of our study participants, and TargetSmart, a voter file consolidator that specialized in collecting and compiling historic voter turnout files at the individual level, we tracked the voter turnout records of each of our study participants. Because we used GfK's panelists, whose personally identifiable information (PII, including each individual participant's full name, birthdate, and full home address) was permanently stored at the company, we sent back the list of our participants' anonymized user IDs preassigned by GfK to the company at the completion of the project. We then had GfK and TargetSmart work together on our behalf to track and match our participants' voter files. GfK then sent back each participant's voting history records with the anonymized user ID, stripping away PII. In this way, we successfully tracked and exactly matched voter files at the individual level even though we did not collect or store our participants' PII. Because we used GfK's own preassigned individual user ID, anonymized for each study participant throughout the study and including ad exposure tracking and survey administrations, we were able to merge voter turnout records with ad exposure, survey responses, and geo-contextual information for each participant.

The 2016 general election turnout records were used for this study to estimate the effect of voter suppression ad exposure on voter turnout. We also used the 2012 general election turnout records for a placebo test to check the robustness of our analysis.

2.5. Data Fusion: Merging with Ads, Surveys, Geographics, and Voter Turnout at the Individual

What we captured by the individual-level ad exposure tracking tool (ad data) was merged with the app user-associated information we collected at the individual level, such as user survey responses (from survey data), geographics (geo-contextual data), and voter turnout records (voter file data). We merged all the different data sets at the individual level with a unique anonymized individual-level identifier (i.e., the merger).

3. Measures

3.1. Identifying Voter Suppression Ads

Following Tova Andrea Wang's definition of voter suppression[10], we define voter suppression as a strategy that demobilizes, discourages, or prevents specific segments of the population from voting. By nature, voter suppression is a strategy that is devised to break the coalition of the opposition and decrease the turnout of voters who are likely to support the opposition. It operates in the interest of a political party. Given this, we identify voter suppression ads based on targets' party ID as well as ad content (messages) and classify different types of voter suppression ads accordingly.

For this study, voter suppression ads are classified into four types: election boycott, deception, third-party candidate promotion, and same-side candidate attack. First, *election boycott (general demobilization)* is the type of ads designed to dampen voters' enthusiasm on elections and discourage voter turnout in general by casting doubt about elections or voting (e.g., "boycott the election", "vote doesn't count"), attacking both sides of the major party candidates (e.g., "neither candidate serves us"). Second, *deception* is deceptive or misleading information about time, manner, and place of voting (e.g., "Democrats vote on Tuesday, Republicans vote on Thursday"; "text your vote to avoid the line"). Third, *third-party candidate promotion* that encourages potential supporters of the opposition to vote for a third-party candidate (e.g., promotion of Bernie Sanders or Jill Stein, especially targeting likely Clinton voters). Fourth, *same-side (candidate) attack* that drives a wedge between potential supporters of the opposition with attack or negative content/tone, especially those attacking the opposition targeting the "weak link" among the potential supporters of the opposition (e.g., ads targeting African Americans that reference Hillary Clinton's 1996 "super predator" comment about African American youth).

We first developed a dictionary that includes the terms frequently used in voter suppression ads based on previous studies[8,11]. Once we "filtered in" potential voter suppression ads by matching voter suppression terms, we took further steps to remove false positives and increase accuracy. First, we removed ads that had commercial sponsors (e.g., Walmart, Amazon) using a commercial sponsor list we compiled. Then, both third-party and official candidate endorsement ads from the candidate themselves (e.g., ads from official Trump campaign sources encouraging users to vote Trump) were also removed by checking the ad sponsors, as well as legitimate GOTV ('get out the vote') ads using the terms in our dictionary because some of the voter suppression terms overlap with GOTV. For example, a legitimate GOTV ad could reference incorrect information about state's ID requirements to correct the misinformation. Next, we adopted an iterative process of drawing random samples from the selected ads, manually examining their relevance to the voter suppression ad types and removing problematic terms from the dictionary. Based on ad content matching, we identified a total of 84,840 potential voter

suppression ads. Finally, after taking into account the ad target's party ID, we refined and identified voter suppression ads by removing anti-Trump ads targeting Democratic voters, anti-Clinton ads targeting Republicans, as well as Gary Johnson, Jill Stein, and third-party candidate promotion ads targeting those who identified their party ID as anything other than "Democrat," "Republican," or "Independent." Table 1 indicates different ad types by target's party ID. Ultimately, we identified and used 59,771 voter suppression ads for this study. We randomly selected 20% of the identified voter suppression ads, and human coders examined whether they were true or false positives. We also examined false negatives by examining random 10% of the non-voter suppression ads. The average accuracy rate (i.e., intercoder reliability, Krippendorff's alpha)[12] was .93 (election boycott .91; deception .98; third party candidate promotion .94; candidate attack .92).

Table S2: Voter suppression ad types

			Third-Candidate Promotion				Same-side Attack	
Target's Party ID	Election Boycott	Deception	Bernie	Johnson	Stein	Other	Anti-Clinton	Anti-Trump
Democrat	742	1306	9182	1047	1685	663	16153	0
Independent	120	172	355	213	176	329	2170	388
Other	106	132	485	0	0	0	1920	364
Republican	501	1057	874	603	555	217	0	2112
Unknown	1749	318	805	308	337	6329	5275	1023

Count of Voter Suppression Ad Type by Target Individual Party ID
"Unknown" observations are where participants did not report their party identification in the survey. These cases were included in the analysis for Figure 1a (bar chart), but not for the HLM and entropy balancing analyses where listwise deletion was applied.

3.2. Exposure to Voter Suppression Ads, Total Ad Exposure, and Total Political Ad Exposure

The exposure to a voter suppression ad was measured at the individual level in two ways: a count variable, i.e., the total number of voter suppression ads each individual was exposed to (median = 6, mean = 15), and a binary indicator of whether the individual was exposed to voter suppression ads. The continuous variable was used to study the targeting pattern of voter suppression ads, whereas the binary indicator was used for the effect analysis. While the count variable would have a higher level of variation that allows us to examine the targeting pattern more precisely with the rich set of covariates, the binary indicator is more suitable for the causal inference framework where "treatment" (exposure) and "control" (non-exposure) are more clearly defined and contrasted in our causal identification and matching method, entropy balancing[13].

We further generate two other online behavior variables: total Facebook ad exposure and overall political ad exposure levels. The total Facebook ad exposure counts the total number of Facebook ads the individual was exposed to (median = 15; zero = 35.7%; between 1 and 14 ads = 14.3%; higher than 14 ads = 50.0%), which functioned as a proxy for overall Facebook use. As a proxy for political interest, we used the total number of political ads across platforms---which included not only Facebook but also Twitter and Google---the individual was exposed to

(median = 6; zero = 56.9%; between 1 and 6 ads = 19.7%; higher than 6 ads = 23.4%). For HLM analysis (**Fig.2b** in the **Main** Text), we took the logarithm of these ad counts and added one to all the values to ensure positive values.

3.3. Socio-Demographics, Political Attitudes, and Behavior Variables

The following variables were obtained from the individual participants' survey responses and used as covariates when we examined targeting patterns and assessed the effects of voter suppression ads. Here we report descriptive statistics of those variables (weighted sample).

The social-demographic variables we use include gender, race, education, income, party ID, and ideology. We recoded race from 5 levels (Caucasian, African-American, Latino/Hispanic, Asian, and Others) to a binary variable (white and nonwhite). We recoded education from 7 levels (8th Grade or less, some high school, high school graduate, some college or associate degree, bachelor's degree, some post-secondary education, graduate or professional degree) to a binary variable (bachelor's degree or higher or not). We recoded ideology from 7 levels (extremely liberal, liberal, slightly liberal, moderate or middle of the road, slightly conservative, conservative, extremely conservative) to 3 categories (liberal, neutral, and conservative).

The following are the final variables that we use in our analyses: gender (female = 50.0%), race (white = 70.3%), education (bachelor's degree or higher = 43.9%), household income (under $20,000 = 13%; between $20,000 and $39,999 = 26.2%; between $40,000 and $59,999 = 20.9%; between $60,000 and $79,999 = 17.1%; between $80,000 and $119,999 = 15.5%; between $120,000 and $249,999 = 9.7%; higher than $250,000 = 1.2%), party ID (Democrat = 50.3%; Republican = 35.4%; Independent = 10.0% and Others = 4.3%), ideology (Liberal = 34.7%, Neutral = 30.7%; Conservative = 34.5%)

The variables that capture individuals' political attitudes include candidate feeling thermometers and issue importance questions. The feeling thermometer measures are questions asking survey participants to indicate how they feel about a candidate on a scale from 0 to 100. A rating between 0 and 50 degrees indicates an unfavorable rating while a rating between 50 and 100 degrees indicates a favorable rating of a candidate. The 14 issue importance questions ask participants how important an issue is to them personally from 1 being "extremely unimportant" to 7 being "extremely important". These include 14 issues (labeled 1 to 14): abortion, climate change, college affordability, gay marriage, global terrorism, gun control, health care, immigration, international trade, jobs and employment, minimum wage, race relations, social security, and tax cuts. We use their original format in our analyses.

Below are the summary statistics for attitude variables. Donald Trump feeling thermometer (range = [0, 100] , mean = 36.7, median = 20.0) ; Hillary Clinton feeling thermometer (range = [0, 100] , mean = 47.8 , median = 51.0); Jill Stein feeling thermometer (range = [0, 100] , mean = 31.1 , median = 29.0); Gary Johnson feeling thermometer (range = [0, 100] , mean = 33.8 , median = 30.0) Abortion issue importance (range = [1,7], mean = 4.7, median = 5.0); climate change issue importance (range = [1,7], mean = 4.7, median = 5.0); college affordability issue importance (range = [1,7], mean = 5.1, median = 6.0); gay marriage issue importance (range = [1,7], mean = 4.0, median = 4.0); global terrorism issue importance (range = [1,7], mean = 5.7, median = 6.0); gun control issue importance (range = [1,7], mean = 5.3, median = 6.0); health care issue importance (range = [1,7], mean = 6.0, median = 6.0); immigration issue importance (range = [1,7], mean = 5.2, median = 5.0); international trade issue

importance (range = [1,7], mean = 4.7, median = 5.0); job and employment issue importance (range = [1,7], mean = 5.9, median = 6.0); minimum wage issue importance (range = [1,7], mean = 5.0, median = 5.0); race relations issue importance (range = [1,7], mean = 5.3, median = 6.0); social security issue importance (range = [1,7], mean = 5.8, median = 6.0); tax cuts issue importance (range = [1,7], mean = 5.2, median = 5.0). Lastly, we have two behavioral variables regarding voting: the 2012 election turnout (self-reported, yes = 79.0%), 2016 election registration status (self-reported, yes = 95.0%).

3.4. Geo-Contextual Information

Battlegrounds. We defined battleground states as those in which the vote margin of the 2016 presidential election was less than 5%. We identified 12 battleground states: Arizona, Colorado, Florida, Georgia, Maine, Michigan, Minnesota, Nevada, New Hampshire, North Carolina, Pennsylvania, and Wisconsin (Battlegrounds = 1; Non-battlegrounds = 0). Among the study participants who completed the baseline survey, 34.4% were from battleground states, and 65.6% were from non-battleground states.

Racial Minority Communities. We operationalized racial minority communities as counties where more than 50% of the residents are nonwhites, following the conventional classification method of the US Census Bureau ("minority county"). County-level population estimates by race ("minority county") were obtained from the American Community Survey 5-year data 2012-2016. Because zip code and counties are not matched one-to-one, it is inevitable to have cases in which same zip code belongs to more than one county. For the analyses using this variable, further weight was calculated for duplicated records generated from the process of matching zip codes to counties by dividing the voting age population of a county by the sum of the voting age population across duplicates. Applying such a weighting scheme is helpful because it reduces zip code-county matching uncertainty, avoiding overestimation of the effect. Then, the cell weights were multiplied to be the total weight for each individual. Besides matching by zip code, we also analyzed the data using FIPS code to match counties for a validity check.

3.5. Voter Turnout: The Individual's Turning Out to Vote

Turnout (Y: Turnout = 1) was observed at the individual level by tracking each study participant's ***actual*** turnout records of the 2016 presidential elections (general election). Most of the past studies have primarily relied on either self-reported surveys for turnout information, which tends to inflate voter turnout rate due to social desirability biases. Other studies adopted aggregate-level actual turnout records, which would not work well for causal inferences that should be based at the individual level[3,14]. Instead, we tracked the study participants' actual turnout records of the 2016 elections. See section 3.4, Voter Turnout Records (Voter File), for details on how we tracked and obtained each individual participant's voter turnout history records.

When matching our participants' data with voter files, we only used cases where *all* the personally identifiable information (PII) *exactly* matched that of the voter files (exact matching). We identified a total of 5,995 individuals exactly matched with voter files. The remaining cases were participants who were not exactly matched or not found in the voter file (unmatched cases). The unmatched cases could be due to no voter turnout or could include missing cases for various

reasons (e.g., missing records at the state level, GfK no longer retains the PII due to the loss of the individuals, such as death or permanent withdrawal). By nature, voter profile data is "presence-only" data — it contains observed positive cases only ($Y_{turnout}$ = 1) with the "unlabeled" in matched cases, or "unmatched" cases, where the individual's actual voter turnout status is unknown. In this case, it is important to discern missing cases from true no turnout ($Y_{turnout}$ = 0) and true turnout ($Y_{turnout}$ =1). As a technique for missing value imputation, we employed the PU-learning technique, which accounts for such data structures[15].

PU-learning is an algorithm built for variable selection and classification for high-dimensional data with presence-only responses. It employs a generalized form of the expectation-maximization (EM) algorithm with regularization to penalize sparsity, enabling it to work effectively with high-dimensional data. Additionally, it leverages sparse matrices and quadratic majorization to scale up the computation.

In the matched sample, labeled cases indicate voter turnout, whereas unlabeled cases could be either "no turnout" or missing at random. We assume that the probability of being unmatched is independent of an individual's turnout status—that is, observations in the unmatched sample represent a random sample of the population with missing responses. To estimate latent individual voter turnout status, we used logistic regression to model the probability of turnout as a function of a number of covariates that theoretically predicted turnout, including age, gender, income, race, voter registration status, and so forth. Assuming that a proportion π of the voters in the matched sample are missing at random, we derived the observed log-likelihood based on the observed label, Z. The Expectation-Maximization (EM) algorithm was then used to maximize this observed log-likelihood to estimate the logistic regression parameters. The turnout probability of each individual in the matched sample was estimated as $\hat{Y} = \hat{P}(Y = 1|x, z)$, representing the predicted probability of turnout conditional on both the covariates (x) and the label (z). For the unmatched sample, the turnout probability was estimated as $\hat{Y} = \hat{P}(Y = 1|x)$, representing the predicted probability of turnout conditional on the covariates (x) alone. For more details on PU-learning protocol, see **Technical Notes**, **PU-Learning** in **SI**.

The PU-learning algorithm includes a hyperparameter, which we calibrated using the Census (ACS) as the benchmark. We set π such that the average predicted turnout probability of our sample (weighted) is closely aligned with the actual voter turnout rate of the voting age population, 61%, as well as those of the key variables, i.e., race (whites vs. nonwhites), states (battlegrounds vs. non-battlegrounds), and counties (minority vs. non-minority counties); thus, the voter turnout rate in our PU-learning outputs to be the same as the actual population voter turnout rate reported by the Census (ACS). As a validity and robustness check, we compared our sample's average voter turnout rates after PU-learning (Sample) with the Census (Census) regarding the key demographics (See Table S3). Table S3 also includes self-reports (Self-Report) as a reference point. While our missing replacement outcomes mirrored the actual turnout in the 2016 presidential election, there was a clear discrepancy between self-reports and the Census for our sample's average voter turnout rate.

Table S3: Average voter turnout rate

Category	Self-Reported	ACS	PULasso
Overall	96%	61%	61%
White	96%	65%	64%
Nonwhite	96%	53%	56%
Battleground	96%	64%	61%
Non-battleground	96%	60%	61%
Minority	98%	55%	58%
Nonminority	96%	61%	62%

Table shows voter participation rates across different demographic and geographic categories using three distinct measurement approaches. The first column presents self-reported voter registration from survey responses. The second column, ACS, shows turnout rates, which provides the official Census Bureau estimates. The third column displays our sample's average turnout rates generated by PU-learning.

4. Analytical Techniques

4.1. Voter Suppression Ad Targeting Patterns

To discover the targeting patterns of voter suppression ads, we first examined the frequency of voter suppression ads by individual level (participant's race; whites vs. nonwhites) and by contextual level (minority vs. nonminority counties and battlegrounds vs. non-battleground). The number of ads was first aggregated into two state-level categories: battlegrounds and non-battlegrounds. Within each category, the voter suppression ads were then grouped by the demographics of the respondents. Finally, voter suppression ads targeting each demographic group were divided into minority-majority counties or not. The denominator was, thus, the total number of voter suppression ads seen by a demographic group (individual-level attributes) within the state category (contextual-level attributes).

$$\text{Proportion of (Voter Supression Ads by Group in County type in State type)} =$$

$$\text{(The Number of Ads by Group in CountyType) / (The Number of Ads by Group in StateType)}$$

$$\text{for State} - \text{Type} \in \backslash\{\text{Battleground, Non} - \text{battleground}\backslash\} \text{ and County} - \text{Type} \in \backslash\{\text{minority, majority}\backslash\}$$

One might be concerned that there might still be disproportional demographic distributions between respondents living in different locations. Furthermore, as some of the previous studies indicated, racial voter suppression (in this case, by racial gerrymandering) might indicate spurious relationships correlated with income and education levels. Accounting for the nesting structure of our data between individuals and counties and to examine the interaction effects between the individual-level (nonwhites) and contextual-level variables (battlegrounds, minority-majority counties) and controlling for the study participants' income, education, and

other potential covariates (party identification, the total number of ads, and the total number of political ads, and the total number of ads that are not political ads), we employed Hierarchical Linear/Nonlinear Modeling (HLM)[16] to examine further targeting patterns of voter suppression ads. The unit of analysis in the model was an individual. The dependent variable was voter suppression exposure, which was defined as the total number of voter suppression ads to which the individual was exposed. Note, HLM does not impose a strict linearity assumption[16,17].

Level-1 Individual-level:

Individuals i nested within counties j:

$$\begin{aligned}\text{VoterSuppressionAdsij} &= \beta0j + \beta1j(\text{Nonwhiteij}) + \beta2j(\text{Incomeij}) + \beta3j(\text{Educationij}) + \beta4j(\text{Democratij}) \\ &+ \beta5j(\text{Republicanij}) + \beta6j(\ln[\text{PoliticalAdExpij} + 1]) + \beta7j(\ln[\text{TotalAdExpij} + 1]) \\ &+ \text{rij}\end{aligned}$$

Where rij is the individual-level residual (random error term). Individuals i nested within counties j

Level-2 Contextual-level (County):

$$\beta0j = \gamma00 + \gamma01(\text{MinorityCountyj}) + \gamma02(\text{Battlegroundj}) + \gamma03(\text{MinorityCountyj}\times\text{Battlegroundj}) + \text{u0j}$$

$$\beta1j = \gamma10 + \gamma11(\text{MinorityCountyj}) + \gamma12(\text{Battlegroundj}) + \gamma13(\text{MinorityCountyj}\times\text{Battlegroundj}) + \text{u1j}$$

Where u0j,u1j are county-level random effects for intercept and the Nonwhite slope, respectively, and slopes allow random variations.

Thus, the final HLM Mixed Model is indicated as below:

$$\begin{aligned}\text{VoterSuppressionAdsij} &= \gamma00 + \gamma01(\text{MinorityCountyj}) + \gamma02(\text{Battlegroundj}) \\ &+ \gamma03(\text{MinorityCountyj} \times \text{Battlegroundj}) + \gamma10(\text{Nonwhiteij}) \\ &+ \gamma11(\text{Nonwhiteij} \times \text{MinorityCountyj}) + \gamma12(\text{Nonwhiteij} \times \text{Battlegroundj}) \\ &+ \gamma13(\text{Nonwhiteij} \times \text{MinorityCountyj} \times \text{Battlegroundj}) + \gamma20(\text{Incomeij}) \\ &+ \gamma30(\text{Educationij}) + \gamma40(\text{Democratij}) + \gamma50(\text{Republicanij}) \\ &+ \gamma60(\ln[\text{PoliticalAdExpij} + 1]) + \gamma70(\ln[\text{TotalAdExpij} + 1]) + \text{u0j} \\ &+ \text{u1j}(\text{Nonwhiteij}) + \text{rij}\end{aligned}$$

4.2. Voter Suppression Effects: Identification and Causal Inference Strategies

To estimate the causal effects of voter suppression ads on voter turnout, we employed a quasi-experimental approach. We examined the (weighted) differences between the "treatment group," (X; exposure to voter suppression ads where Exposure = 1, No Exposure = 0) and "control group" or "counterfactual," (no exposure to voter suppression ads), in terms of predicted probability of voter turnout while the distributions of the covariates being the same between the treatment and counterfactual. The unit of analysis here is an individual, and the outcome variable is the individual's predicted turnout probability (Y: Turnout =1 ~ No turnout = 0).

Identification, Matching, and Balancing. To adequately infer the treatment effects for identification, matching, and balancing strategies, we employed Hainmueller's *entropy balancing*[13] with double selection. We first identified all the possible covariates by employing a *double selection*[18,19]---running two Lasso regression models predicting (1) turnout and (2) voter suppression exposure with all the variables that are related to either turnout or ad exposure[19]. We began with 44 variables and retained 35 statistically or theoretically significant ones.

The Lasso regression serves two purposes. First, it reduces the chance of omitted variable bias compared to running one regression of turnout on exposure and other covariates. Second, Lasso performs well for variable selection given our large number of variables. The variables selected as covariates include: education, income, 2012 turnout, 2016 voting registration, party identification, ideology, gender, age, race, the total number of ads the individual received, the total number of political ads the individual received (excluding voter suppression ads), issue importance of 14 issues (see Measures, section 4.3.), and candidate feeling thermometers. Together, the double selection procedure, lasso shrinkage, and entropy balancing's doubly robust property (more details below) makes our estimates robust and conservative.

Entropy balancing[13] was then used to assign weights to the control group respondents such that the distribution of covariates between control and treated respondents would be the same. It is a pre-analysis processing method for causal inferences based on observational data with a binary treatment and achieves covariate balance through reweighting. This approach has several merits for our study. First, it works with a large set of covariates while keeping all the data, whereas exact matching or nearest neighbor matching discards unmatched cases. Thus, it is less likely to run into statistical power issues. Second, compared to other methods, including propensity score methods[20], entropy balancing frees us from repeatedly specifying the propensity score model and examining covariate balance by directly including covariate balance in the estimation procedure. While propensity score methods are extremely popular for causal inferences, for this reason, some researchers, such as Gary King and Richard Nielsen, argue propensity scores should not be used as they often produce the opposite of the goals, increasing imbalance, inefficiency, model dependence, and bias[21]. Third, entropy balancing has the property of double robustness[22]. The estimator is consistent for the Population Average Treatment effect for the Treated (PATT) if at least one of the two models is correctly specified[22]. Lastly, as a covariate distribution-balancing model that reweights covariates to match moments across treatment and control groups, entropy balancing is agnostic about the functional form of the outcome relationship. Entropy balancing helps avoid model dependence as it is not based on the specification of outcome models. Thus, no linearity assumption is required regarding how outcomes are related to the treatment[13]. For details of the method, refer to Hainmueller 2012[13] and Zhao and Percival's 2016[22] recent discussion[23,24]. This also confirms the robustness of entropy balancing applied to a wide range of statistical problems, including nonlinear interaction models. At the end of this document, we include **Technical Notes, Identification and Balance Strategies for Causal Analysis** detailing our causal inference protocols using entropy balancing.

Convergence check. After applying entropy balancing, no difference in the mean should be observed between the treatment (Exposure) and control (Non-exposure) groups in terms of the selected covariates. Figure S2 shows the mean difference comparison between before and after we apply entropy balancing weights to the control variables. With the weights, the adjusted mean differences between the treatment and control groups become all zeros. See **Fig.S1**.

Fig.S1: Mean difference between the exposure and nonexposure before-after entropy balancing

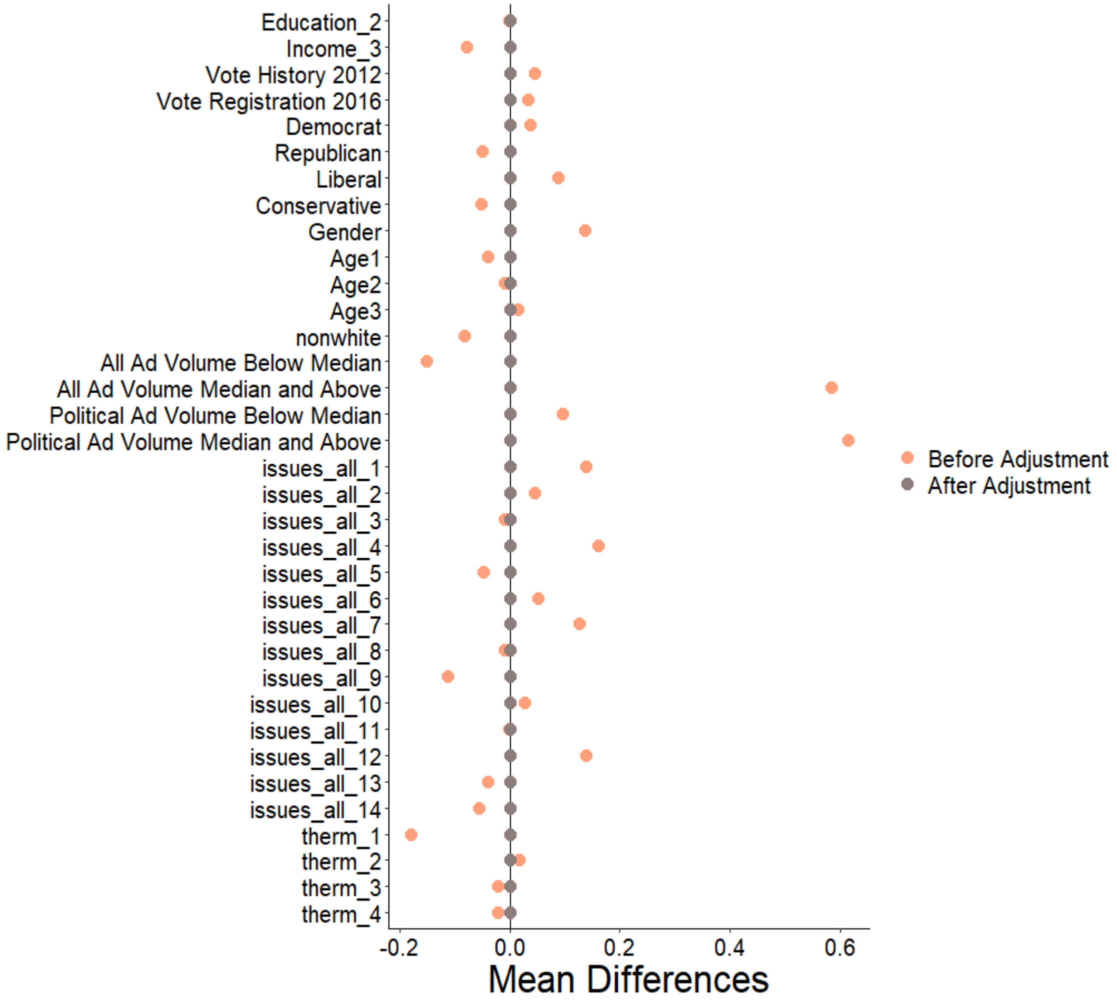

Standardized mean differences between treatment (exposed to voter suppression ads) and control (not exposed) groups across 35 covariates, before and after entropy balancing adjustment. Salmon-colored points represent differences before adjustment, while gray points show differences after adjustment. The balancing procedure successfully reduced all covariate differences to approximately zero, as indicated by the aligned gray points at the zero line. Covariates include demographic characteristics (education, income, gender, age, race), political variables (vote history, registration, party identification, ideology), ad exposure measures (total and political ad volumes), issue positions on 14 policy areas, and feeling thermometer ratings for presidential candidates. This balance assessment demonstrates the effectiveness of the entropy balancing procedure in creating comparable treatment and control groups for causal inference. After entropy balancing, the two groups were perfectly balanced, indicating no difference in terms of the distribution of covariates. Thus, any difference in voter turnout between the two groups is due to the Treatment (exposure to voter suppression).

Effect Assessment. Once we obtained the entropy balancing weights, we applied the entropy balancing weights to effect models. The average treatment effect (ATE) would be simply the mean differences in voter turnout probability between the exposure and non-exposure groups. The ATE result can be found in **Main Text, Fig.2**, with violin charts in a full scale here, **Fig.S2**

Fig.S2: Average treatment effects (ATE) of voter suppression exposure on voter turnout

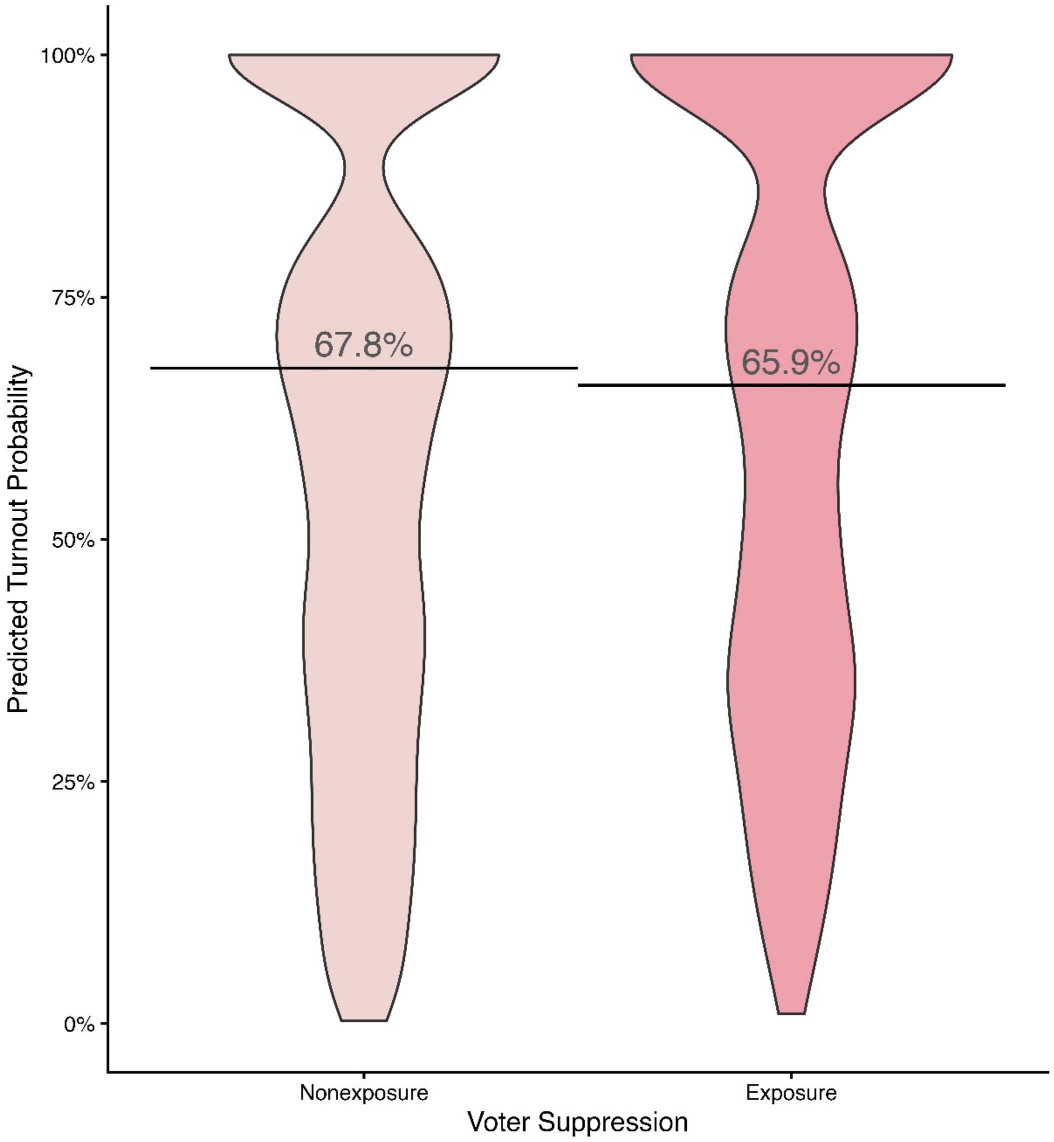

Distribution of turnout probabilities for those not exposed and those exposed to at least one voter suppression advertisement. The two horizontal lines indicate the average voter turnout for the two groups (turnout =1, no turnout=0). The group not exposed to voter suppression ads had an average turnout probability of 67.7%. The exposed group has an average turnout 65.9%, representing a significant decrease of 0.0185 points (1.85%) (b=-0.019, t(8546)=-2.72, p=0.006). Model fit: $F(1,8546)=7.42$, p=0.006, $R^2=0.0009$.

We also assessed the heterogeneous treatment effects (HTEs) to assess how different target audience groups responded to the treatment differently. We examined the differences between non-whites and whites; battleground and non-battleground states; and minority (population of racial minorities is larger than 50% of the total population) and nonminority counties. For each moderating variable, we divided the sample into corresponding subsamples (e.g., nonwhites vs. whites) and repeated the entropy balancing process separately for each group to ensure that the covariates would be as balanced as possible within each group. After rebalancing, we merged the subgroups back and introduced interaction terms between the moderating variables and the main treatment variable in the linear model to estimate the HTEs. For details on our causal inference protocol, see **Technical Notes, Identification and Balance Strategies for Causal Analysis**.

5. Robustness Checks

5.1. Treatment Effects with Different Control Groups (Counterfactuals)

To check the robustness of the results, we tested the treatment effects with different types of control groups (counterfactuals).

5.1.1. Average Treatment Effects with Different Counterfactuals

The primary treatment effect test described in the Main Text compares those exposed to a voter suppression ad (Treatment: voter suppression exposure = 1) and those not exposed to any voter suppression ads (voter suppression exposure = 0), the control group, Counterfactual A. In addition, we compared the treatment group with a different control group, Counterfactual B: those who were not exposed to voter suppression ads, but exposed to *other political ads* (voter suppression = 0, but exposure to non-voter suppression political ads = 1); or, another control group, Counterfactual group C: those who were not exposed to *any* political ads (voter suppression exposure = 0 and political ad exposure = 0). We expected that the Treatment group (voter suppression exposure) would still show decreased voter turnout compared to Counterfactual B even though the treatment against Counterfactual B is an even stricter test than Counterfactual A. Compared to Counterfactual C, we expected no effect.

Or, since voter suppression is still political by nature, we would observe an opposite effect when compared to exposure to no political ads. Then, to further differentiate the effects of voter suppression and the effects of non-voter suppression political ads, we set Counterfactual B as a new treatment (Treatment 2: voter suppression exposure = 0, but exposure to non-voter suppression political ads =1) and used Counterfactual C as a control. The Treatment 2 effects would indicate the effects of non-voter suppression political ads. Here, we expected to observe a stark contrast between the effects of the Treatment group, voter suppression exposure group, and those of Treatment 2 group, non-voter suppression political ad exposure group. We used the same matching and entropy balancing strategies with the same covariates, where applicable.

These additional tests (**Fig.S3**) confirm the robustness of the treatment effects described in the Main Text (**Fig.3** in **Main Text**; also a. in **Fig.S3**). Those exposed to voter suppression ads still exhibited a lower turnout level than those exposed to other political ads, Counterfactual B (b. in **Fig.S3**). The exposure to voter suppression did not appear to depress turnout when compared to the average turnout rate of those who were not exposed to any political ads at all, Counterfactual C. Still, considering the mobilizing effects of non-voter suppression political ads

(Treatment 2, d. in **Fig.S3**), the findings indeed are consistent---Voter suppression exposure is distinct from exposure to other political ads and generates unique effects of voter turnout depression.

Fig.S3: Average Treatment effects with different counterfactuals

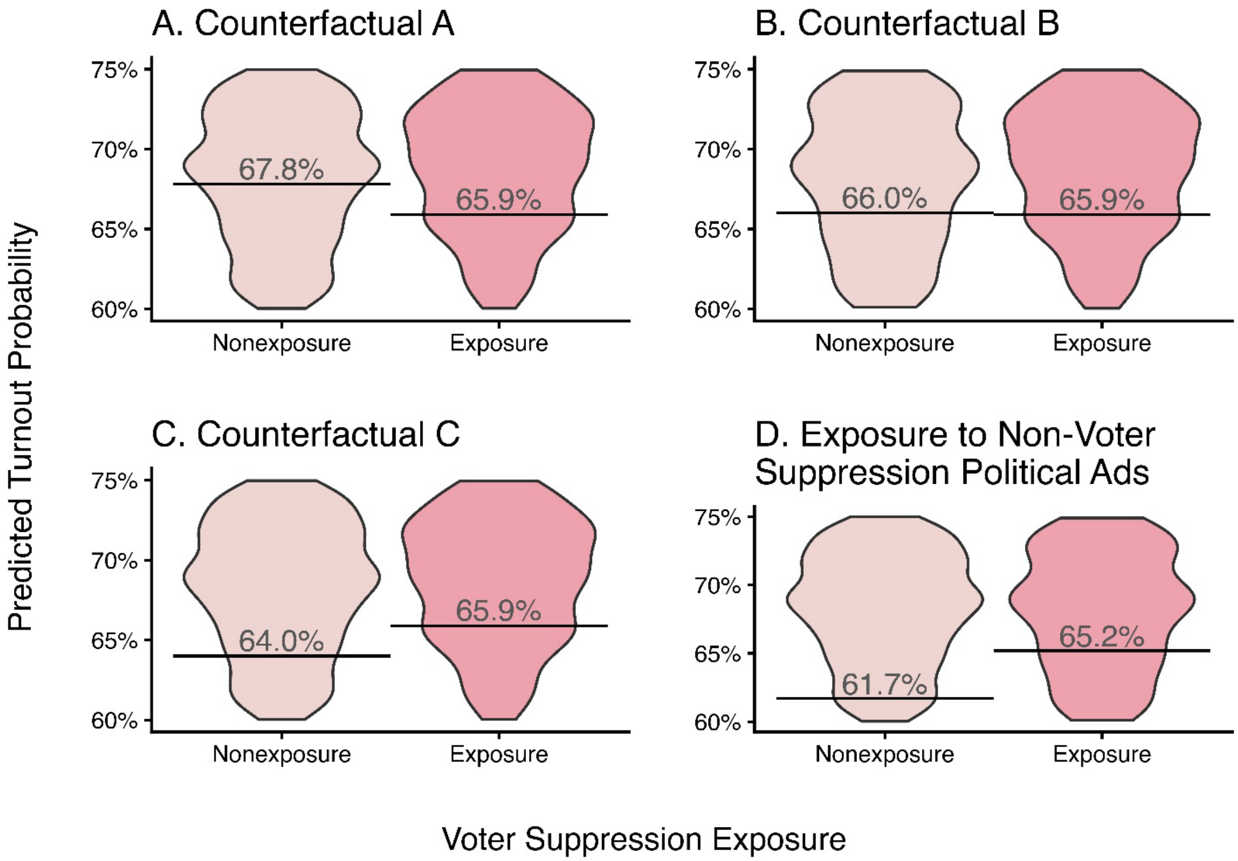

Predicted turnout probabilities comparing voters exposed (Treatment) and not exposed (Control/Counterfactual) to voter suppression ads across different specifications of counterfactuals. Violin plots display the distribution of predicted probabilities, with horizontal lines indicating group means. Panel A shows the baseline model F(1,8546)=7.42, p=0.006, R^2=0.0009, RSE=0.228, with mean probabilities of 67.7% for non-exposed and 65.9% for exposed voters. Panel B presents the first counterfactual using alternative matching (F(1,4046)=0.02, p=0.901, R^2<0.001, RSE=0.331), with means of 66.0% and 65.9%. Panel C shows a second counterfactual specification (F(1,6743)=6.46, p=0.011, R^2=0.001, RSE=0.251), with means of 64.0% and 65.9%. Panel D examines exposure to non-voter suppression political ads as a placebo test (F(1,6301)=19.62, p<0.001, R^2=0.003, RSE=0.232), with means of 61.7% and 65.2%. All models are weighted linear regressions with robust standard errors.

5.1.2. HTE with Different Counterfactuals
: Conditional Treatment Effects (CTE) with Different Subgroups

We also conducted a robustness check on the heterogeneous treatment effects (HTE) with different counterfactuals by performing conditional treatment effect (CTE) analysis across

various subgroups. First, we estimated the effects of voter suppression on the turnout of the targeted segments, i.e., nonwhite voters in minority counties of battlegrounds only. We compared the turnout of the targeted segments—in this case, nonwhites in minority counties of battlegrounds—who were exposed to voter suppression and that of the same targeted segments who were not exposed to any voter suppression ads (counterfactual-d, missed exposure group). This test will provide us with a better understanding of how robust the effects of voter suppression are among the targeted segments of the population. For comparison, we also compared the turnout of the targeted exposed group to that of another group, namely those who were exposed to voter suppression ads but did not fit the targeted segment, specifically white voters in non-minority populations of non-battleground states (counterfactual-e, mistargeted group). These subgroup comparisons would give us an idea of how strong geo-racial targeting would be even among those who were exposed to voter suppression ads.

Fig.S4: HTEs with Counterfactuals: Conditional Treatment Effects with Different Subgroups

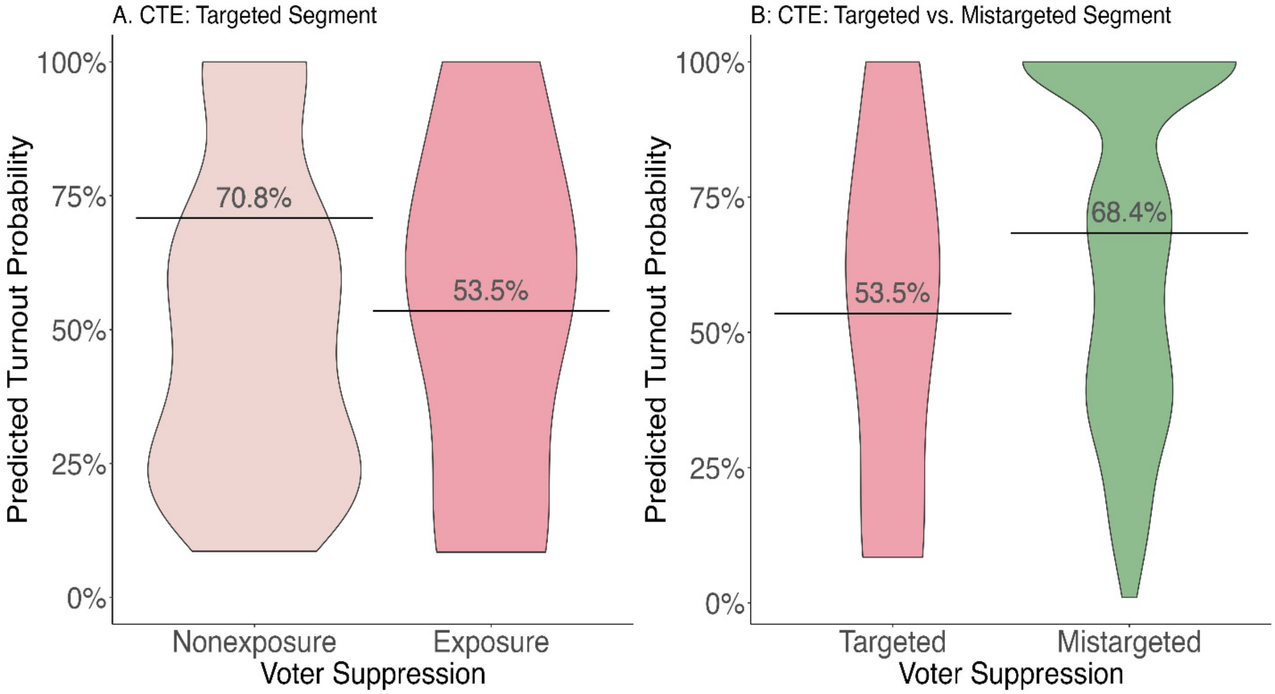

Predicted turnout probabilities comparing the treatment effect of voter suppression exposure conditioned upon specific subgroups. Violin plots display the distribution of predicted probabilities, with horizontal lines indicating group means. A. shows the predicted turnout probability among targeted segments only, i.e., nonwhites residing in minority counties of battleground states. CTE (differences between exposure and non-exposure) is 17.3%. B. shows the predicted turnout probabilities between the targeted segments (nonwhites in minority counties in battlegrounds who were exposed to voter suppression) and the mistargeted group (whites in nonminority counties in non-battlegrounds). The mean difference is 14.9%. CTEs were conducted with entropy balancing with subsamples.

The results show that the turnout rate was significantly lower—in fact, the lowest---among the targeted exposed group when compared to the missed exposed group (counterfactual-d) or the mistargeted group (counterfactual-e), confirming the robust heterogeneous effects of the targeted voter suppression ad exposure on voter turnout.

5.2. Sensitivity Analysis

One might argue that the causal effects might have been influenced by unobserved or unmeasured covariates that were not taken into account in the identification. To test how sensitive our results are to omitted variable bias, we employed sensitivity analysis, which calculates the amount of confounding needed to render our treatment effect insignificant and compare the threshold to our control variables[25,26]. The idea is to gauge the possibility of omitted variables that bias the results. Compared to the existing covariates, unobserved variables are likely to have a smaller confounding effect. Thus, if even the existing covariates do not have enough confounding effects to change the results, the estimates are unlikely to suffer from omitted variable bias. The x-axis measures the partial correlations between the confounders we control in entropy balancing and predicted turnout probability, whereas the y-axis is the partial correlation between the confounders and the treatment, in our case, exposure to voter suppression ads.

We obtained the partial correlations by regressing the predicted turnout probability on the 35 confounders (all the controlled covariates in our analysis) and each potential unobserved confounder correlated with each covariate on the treatment and the rest of the confounders. The multiplication of the two is then a confounding effect if a covariate is not controlled. Based on that, the contour lines are the hypothetical effect estimate by subtracting the bias from the treatment effect value, and the red line ("Reject the null at p <.05") is the threshold for a minimum significant non-zero effect.

The sensitivity analysis (**Fig.S5**) clearly shows that it is highly unlikely that unobserved confounders explain the causal effects analyses. The Jill Stein feeling thermometer (therm 4) had the highest level of correlations with the exposure to voter suppression ads, and the most correlated variable to turnout probability was previous turnout (vote 2012). Considering that even these two covariates, or the potential confounders correlated to the covariates, which might pose the biggest threats to our effect estimates, were only moderately correlated and significantly below the threshold. Most of the variables have virtually zero confounding influence. Thus, even the most promising confounders related to the feeling thermometer and the 2012 voting history would not challenge the robustness of our causal inferences. Given the high number of our covariates and both theoretical and empirical variable selection processes, the results from the sensitivity analysis indicate our causal inference analysis is robust. Thus, it is highly unlikely that our causal effect analysis is explained by unfound or unmeasured variables.

Fig.S5: Sensitivity analysis

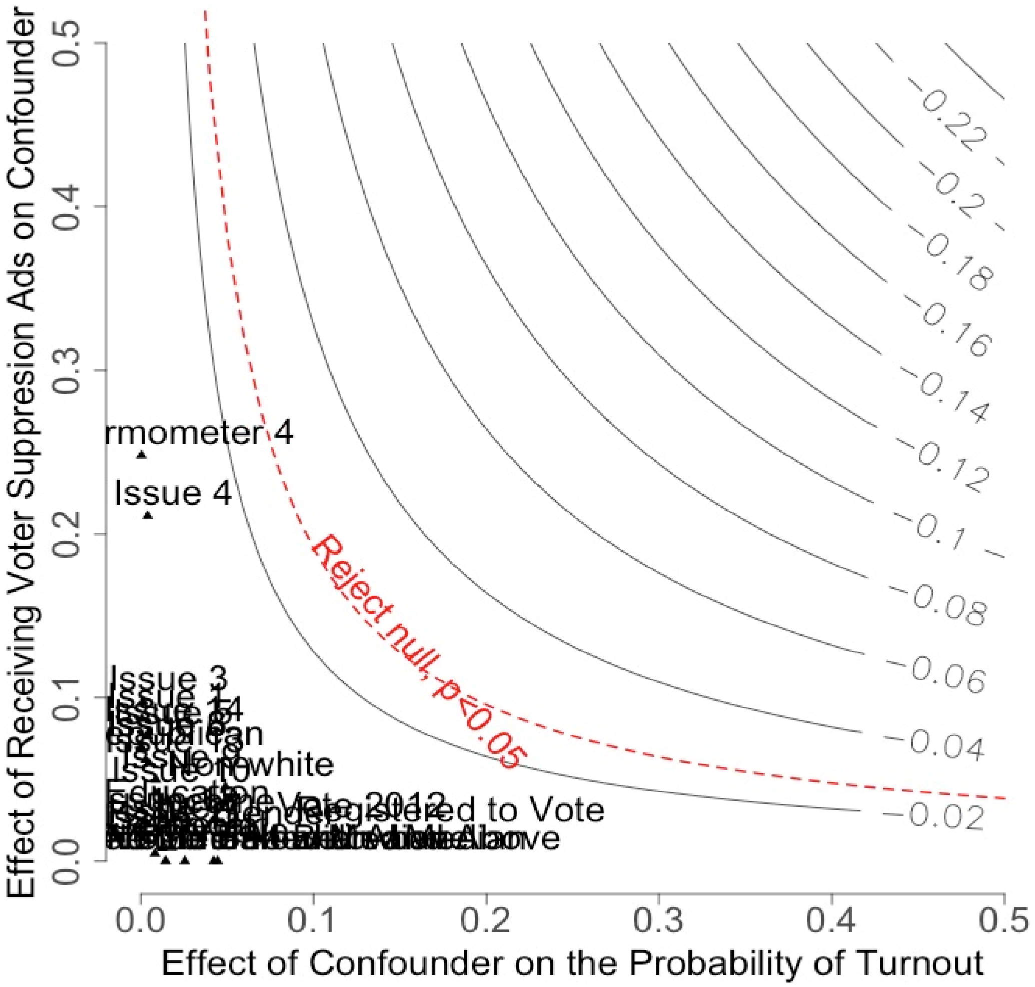

Sensitivity analysis for the effect of voter suppression ads on turnout probability. Contour lines represent the bias-adjusted treatment effect size under different combinations of confounding. The x-axis shows the hypothetical effect of an unmeasured confounder on turnout probability, while the y-axis shows the effects of exposure to voter suppression ads on the confounder. The dashed red line indicates the threshold where the treatment effect would no longer be statistically significant ($p>0.05$). Black triangles represent observed covariates plotted according to their relationship with treatment assignment and outcome, with labels indicating the covariate name. Contour values indicate the magnitude of the treatment effect after accounting for potential confounding at each point in the parameter space. Most of the covariates have nearly zero confounding influence. The potential bias contributions ($|\gamma \times \alpha|$) are shown in this figure, but also listed as below: Age=0.0018; Education 0.0003; Income 0.0006; Vote 2012 0.0022; Gender 0.0009; Democrat 0.0001; Republican 0.0002; Conservative 0.0000; Liberal 0.0001; All Ad Volume Below Median 0.0000; All Ad Volume Median and Above 0.0000; All Ad Volume Zero –; Political Ad Volume Below Median 0.0000; Political Ad Volume Median and Above 0.0000; Political Ad Volume Zero –; Registered to Vote 0.0024; Non-white 0.0022; Abortion Importance 0.0001; Climate Change Importance 0.0000; College Affordability Importance 0.0001; Gay Marriage Importance 0.0009; Global Terrorism Importance 0.0003; Gun Control Importance 0.0000; Health Care Importance 0.0002; Immigration Importance 0.0000; International Trade Importance 0.0004; Jobs & Employment Importance 0.0004; Minimum Wage Importance 0.0001; Race Relations Importance 0.0000; Social Security Importance 0.0003; Tax Cuts Importance 0.0004; Donald Trump Thermometer 0.0006; Hillary Clinton Thermometer 0.0001; Jill Stein Thermometer 0.0006; Gary Johnson Thermometer 0.0001.

5.3. Placebo Analyses

5.3.1 False Shock Test

As a placebo test, we chose an "omitted" variable that should be unrelated to any of the treatment or outcome variables, such as the operating system of the participant's device, which was tagged in our data as meta-information in our data collection. If we use the operating system (Windows =1, others = 0) as a "treatment," we should not observe the same effect on voter turnout. If it contributes to explaining the relationship between voter suppression exposure and turnout, it would indicate unobserved variables that might have contributed to the observed effects. While we used the same identification and balancing strategies, we found no significant effect of this "false shock" on voter turnout (b =.007, t-value =.997, p =.319, Cohen's d =.022).

5.3.2. Prediction for the 2012 Voter Turnout

We also modeled the effect of voter suppression exposure predicting the 2012 voter turnout. Since the exposure to voter suppression was measured in 2016, it should not produce the same decreases in voter turnout. We used the exact identification and balancing strategies with the same covariates (except that "2008 turnout," instead of the 2012 turnout, was utilized). We obtained the participants' 2012 and 2008 turnout records from the same voter file. With no missing replacement (because our PU-learning model for the 2016 turnout would not be adequate for the previous years' turnout models, theoretically), we tested the treatment, exposure to voter suppression in 2016, on the 2012 voter turnout. We found that voter suppression did not decrease voter turnout in 2012, although it was positively associated (b = .03, t = 2.14, p = .03, Cohen's d = .062). The positive relationship, perhaps, was because voter suppression targeting was a function of past turnout.

5.4. Effect Analysis with Different Identification and Balancing Strategies

While entropy balancing was most adequate, and the identification and balancing were completed successfully, here we present additional analysis with different identification and balancing strategies. If our findings are robust, the patterns in the results should be consistent even when different identification and strategies are applied. We employed two other identification strategies, exact matching and full matching with covariate balancing propensity score (CBPS).

5.4.1. Exact Matching

Exact matching is the most stringent and conservative method in causal inference.[27,28] It identifies exactly matched pairs where all the covariate values are identical; thus, the only difference is the treatment itself. It provides the most robust basis for causal inferences. However, because it eliminates all the unmatched cases, it often has an insufficient number of cases for statistical power.

With exact matching, we still found the *same* pattern---exposure to voter suppression did decrease voter turnout by 5.7%, although it was not statistically significant due to insufficient statistical power (p = .43).

5.4.2. Full Matching with Covariate Balancing Propensity Score (CBPS)

Full matching creates pairs or clusters of treated and control units, where overall differences of covariates are reduced[29]. Unlike exact matching, full matching does not require precise matches on all the covariates, but rather "optimizes" the balances in covariates across treated and control groups as a whole. This method is particularly useful when exact matches are rare due to the high dimensionality of data.

We used propensity scores for covariate optimization, specifically full matching with CBPS. The results are consistent---voter suppression exposure decreased turnout (1.9% decrease, $p = .25$). Unfortunately, the distributions of propensity scores were quite unbalanced, rendering less desirable outcomes statistically insignificant.

Supplemental Analysis and Its Results

1. Unpacking Targeting Patterns

We illustrate the targeting patterns of voter suppression ads in **Fig. 2**a (descriptive bar charts) and **Fig. 2**b (tree plots based on the HLM full model) of the Main Text. It indicates voter suppression ads are concentrated in minority counties, especially nonwhites in battleground states. As Fig.2a shows a conditional marginal probability, conditioned on minority counties, here we show alternative figures conditioned on battlegrounds (**Fig.S6**a) and conditioned on nonwhites (**Fig.S6**b)

Fig.S6: Targeting Patterns: Voter Suppression Ads in Battlegrounds and Nonwhites

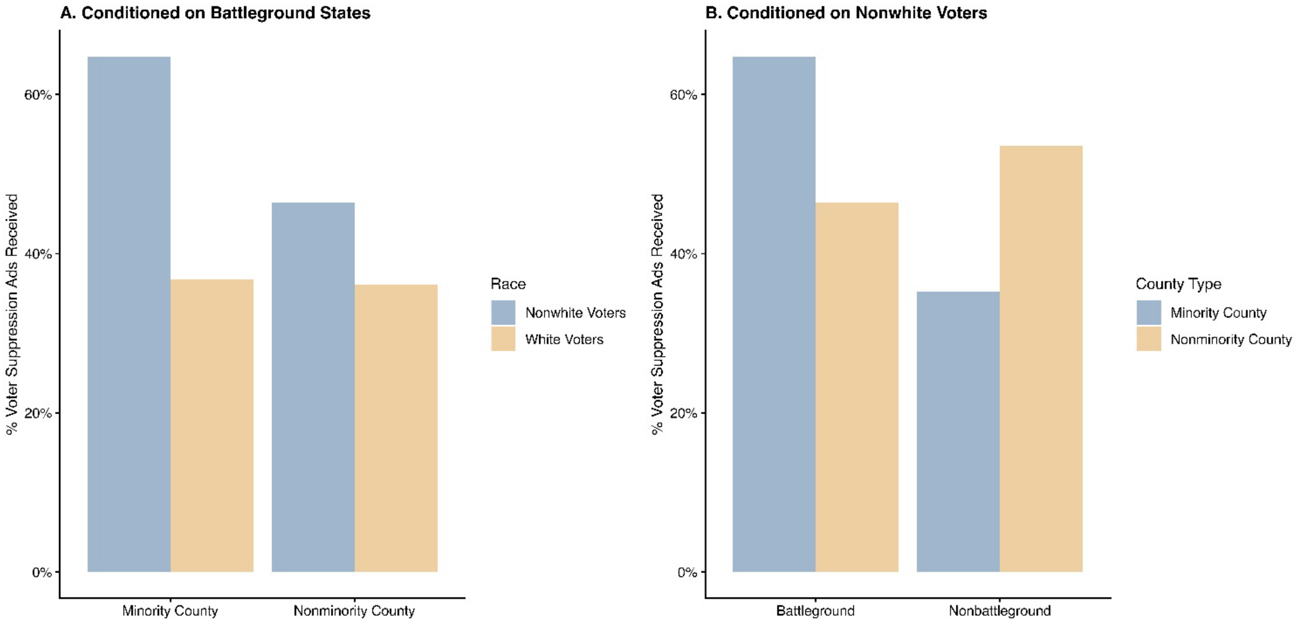

2. Unpacking Heterogeneous Treatment Effects

Our heterogeneous treatment effects (HTEs) are subgroup comparisons based on the combinations of the four attributes: treatment (VS: voter suppression exposure vs. non-exposure) x race (non-whites vs. whites) x state (battleground vs. non-battleground) x county (minority vs. nonminority). The descriptive statistics, the estimated average of turnout rates and the standard errors, for each group are summarized in **Table S4.**

Table S4: Voter Turnout Rates by Group

Group				Entire Sample (Population)	
Exposure	Battleground	Minority	Group	Turnout rate (population EST)	SE
Exposure	Battleground	Minority	Nonwhite	53.5%	7.2%
			White	46.0%	3.8%
		Nonminority	Nonwhite	57.7%	2.2%
			White	64.8%	0.8%
	Nonbattleground	Minority	Nonwhite	67.0%	7.6%
			White	67.5%	3.3%
		Nonminority	Nonwhite	63.6%	1.7%
			White	68.4%	0.7%
Nonexposure	Battleground	Minority	Nonwhite	70.8%	6.7%
			White	48.9%	4.4%
		Nonminority	Nonwhite	64.2%	2.2%
			White	70.4%	0.9%
	Nonbattleground	Minority	Nonwhite	79.1%	6.8%
			White	63.5%	5.3%
		Nonminority	Nonwhite	62.1%	1.7%
			White	67.7%	0.7%

We balanced the treatment (exposure to voter suppression, VS = 1) and the control group (VS = 0) with entropy balancing in terms of all of the35 covariates, but also included key moderating variables, nonwhite, minority, and battlegrounds, and the interaction terms between the treatment and the moderators. Entropy balancing weights (as well as sample weights) are applied to the models (**Table S5a**). We present the heterogeneous treatment effects (HTE) results in the **Main Text**, **Fig.4**, which indicate the geo-racial targeting effects of voter suppression on voter turnout. We note that our subgroup comparisons generate relatively large error bars (standard errors) for specific subgroups, where the cell size is small. While it might indicate a relatively low level of precision in statistical inference (thus a large p-value), our point estimates are relatively robust; thus, differential effects should not be dismissed. The HTEs should be evaluated holistically, taking into account effect size. We report the effect size of (conditional) treatment effects in the Main Text. We also employed entropy balancing with multiplicative split samples: first split the sample into subsamples and applied entropy balancing between the treatment (VS = 1) and the control (VS = 0) within a sub sample (e.g., whites, nonwhites, nonwhites in battlegrounds, etc.). **Fig.S7** presents the results. We repeated the procedure for each of the subsamples where convergence between the treatment and control in terms of covariates occurs. **Table S5b** presents the results with multiplicative split sample reweighting.

Table S5a: Heterogeneous treatment effects: Treatment EB weighted stepwise regression models

	Predicted Voter Turnout							
	(1)	(2)	(3)	(4)	(5)	(6)	(7)	(8)
VS	−0.02**	−0.02*	0.01	−0.02*	0.01	−0.02*	0.01	0.01
	(0.01)	(0.01)	(0.01)	(0.01)	(0.01)	(0.01)	(0.01)	(0.01)
NW		−0.05**			−0.05*	−0.06***		−0.06**
		(0.01)			(0.02)	(0.01)		(0.02)
VS×NW		−0.01			−0.001	0.003		0.01
		(0.02)			(0.03)	(0.02)		(0.03)
BG			0.02		0.02		0.02*	0.03*
			(0.01)		(0.01)		(0.01)	(0.01)
VS×BG			−0.06***		−0.06***		−0.06***	−0.06***
			(0.01)		(0.02)		(0.01)	(0.02)
Min				−0.06*		−0.14***	0.02	−0.04
				(0.03)		(0.03)	(0.04)	(0.05)
VS×Min				−0.01		0.05	−0.03	0.03
				(0.04)		(0.04)	(0.05)	(0.06)
NW×BG					−0.001			−0.005
					(0.03)			(0.03)
VS×NW×BG					−0.02			−0.02
					(0.04)			(0.04)
NW×Min						0.26***		0.21*
						(0.06)		(0.09)
VS×NW×Min						−0.19*		−0.17
						(0.08)		(0.12)
BG×Min							−0.16**	−0.17*
							(0.06)	(0.07)
VS×BG×Min							0.005	−0.01
							(0.07)	(0.09)
NW×BG×Min								0.07
								(0.12)
VS×NW×BG×Min								0.03
								(0.17)
Constant	0.68***	0.68***	0.67***	0.68***	0.68***	0.69***	0.67***	0.68***
	(0.005)	(0.01)	(0.01)	(0.005)	(0.01)	(0.01)	(0.01)	(0.01)
Observations	8,548	8,548	8,548	8,548	8,548	8,548	8,548	8,548
R^2	0.001	0.004	0.004	0.003	0.01	0.01	0.01	0.01
Adjusted R^2	0.001	0.003	0.004	0.002	0.01	0.01	0.01	0.01

* p<0.05, ** p<0.01, and *** p<0.001

Table presents stepwise regression models predicting voter turnout, with eight different model specifications (1-8). Coefficients represent relationships between voter turnout, treatment (VS), and various predictors: voter suppression ad exposure (VS= exposure =1), voter race (NW = non-white), geographic location (BG = battleground state), and community composition (Min = minority county). Models progressively incorporate interaction terms, denoted by × between variables (e.g., VS×NW represents the interaction between voter suppression exposure and non-white status). For each predictor, the main number shows the coefficient estimate, with standard errors in parentheses below. The bottom panel reports model fit statistics. All models use entropy-balanced weights with base weights to account for pre-treatment covariate imbalances.

Table S5b: Heterogeneous treatment effects: Multiple split-sample EB reweighted models

	Predicted Voter Turnout							
	(1)	(2)	(3)	(4)	(5)	(6)	(7)	(8)
VS	−0.02**	−0.02**	0.01	−0.02*	−0.01	−0.05***	−0.01	−0.01
	(0.01)	(0.01)	(0.01)	(0.01)	(0.01)	(0.01)	(0.01)	(0.03)
Non-White		−0.11***			0.01	−0.08***		0.05*
		(0.01)			(0.02)	(0.01)		(0.02)
VS×NW		0.06**			−0.06	0.02		−0.09
		(0.02)			(0.04)	(0.03)		(0.09)
BG			0.01		0.05***		0.06***	0.15***
			(0.01)		(0.01)		(0.01)	(0.01)
VS×BG			−0.05***		−0.10***		−0.09***	−0.19***
			(0.01)		(0.02)		(0.02)	(0.05)
Min				−0.06*		−0.32***	−0.02	−0.19
				(0.03)		(0.03)	(0.05)	(0.11)
VS×Min				−0.01		0.23***	0.01	0.18
				(0.04)		(0.05)	(0.08)	(0.19)
NW×BG					−0.20***			−0.34***
					(0.02)			(0.03)
VS×NW×BG					0.18**			0.32*
					(0.06)			(0.14)
NW×Min						0.47***		0.25
						(0.05)		(0.14)
VS×NW×Min						−0.40***		−0.21
						(0.11)		(0.42)
BG×Min							−0.26***	−0.34**
							(0.06)	(0.12)
VS×BG×Min							0.10	0.16
							(0.10)	(0.27)
NW×BG×Min								0.42*
								(0.17)
VS×NW×BG×Min								−0.32
								(0.58)
Constant	0.68***	0.69***	0.67***	0.68***	0.69***	0.72***	0.68***	0.69***
	(0.005)	(0.01)	(0.01)	(0.005)	(0.01)	(0.004)	(0.01)	(0.005)
Observations	8,548	8,548	8,548	8,548	8,548	8,548	8,548	8,548
R^2	0.001	0.01	0.003	0.003	0.02	0.02	0.02	0.08
Adjusted R^2	0.001	0.01	0.003	0.002	0.02	0.02	0.02	0.08

* p<0.05, ** p<0.01, and *** p<0.001

Table presents regression models predicting voter turnout with multiple split samples where entropy balancing re-weighted for each split sample, and multiplicative reweights applied with eight different model specifications (1-8). Coefficients represent relationships between voter turnout and various predictors: voter suppression ad exposure (VS), voter race (NW = non-white), geographic location (BG = battleground state), and community composition (Min = minority county). Models progressively incorporate interaction terms, denoted by × between variables (e.g., VS×NW represents the interaction between voter suppression exposure and non-white status). For each predictor, the main number shows the coefficient estimate, with standard errors in parentheses below. The bottom panel reports model fit statistics. Each model uses multiplicative entropy balancing reweights that combine weights across different subgroup analyses.

Fig.S7: Conditional Average Treatment Effects (Split-Sample Reweighting)

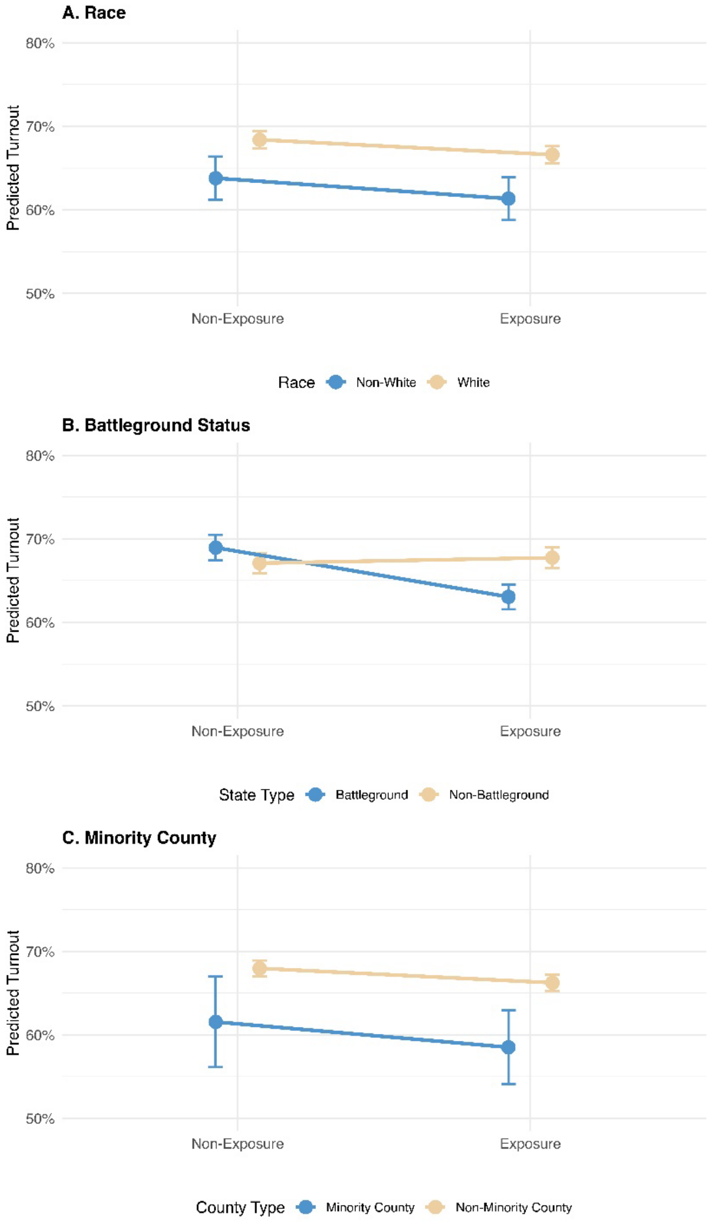

Predicted voter turnout between voter suppression exposure vs. nonexposure in: **a**, Nonwhite and white respondents; **b**, battleground and non-battleground state residents; and **c**, minority county and nonminority county residents. This figure is based on three separate two-way interaction models using subsample-split balanced data, where treatment and control groups are reweighted via subsample-specific entropy balancing to achieve covariate balance. Points indicate the unstandardized predicted turnout estimates, and error bars describe the 95% CI based on weighted linear regression models. The X-axis shows exposure status (Non-Exposure vs. Exposure). The y-axis shows the predicted turnout probability. Panel **a**: Model includes voter suppression exposure, race (nonwhite vs. white), and their interaction term; blue points/lines represent Non-White respondents, tan points/lines represent White respondents; $F(3,8544)=10.78$, $p<.001$, $R^2=0.0038$, RSE=0.228. Panel **b**: Model includes voter suppression exposure, battleground state status (battleground vs. non-battleground), and their interaction term; blue points/lines represent Battleground state residents, tan points/lines represent Non-Battleground state residents; $F(3,8544)=11.32$, $p<.001$, $R^2=0.0040$, RSE=0.228. Panel **c**: Model includes voter suppression exposure, minority county status (minority vs. nonminority), and their interaction term; blue points/lines represent Minority County residents, tan points/lines represent Non-Minority County residents; $F(3,8544)=8.04$, $p<.001$, $R^2=0.0028$, RSE=0.228.

3. Effects of Different Voter Suppression Ad Types

We also analyzed the voter suppression ad effects by different types of voter suppression ads (see **Table S2**). Overall, we found statistically significant differential effects of ad types, confirming the utility of (re)conceptualization of voter suppression in the digital era. Given our interest in particular types of geo-racial targeting, nonwhites in battlegrounds or minority counties, here we focus on differential patterns most relevant to such segments (**Fig.S8**).

Fig.S8: Heterogeneous effects by different ad types

A. Election Boycott

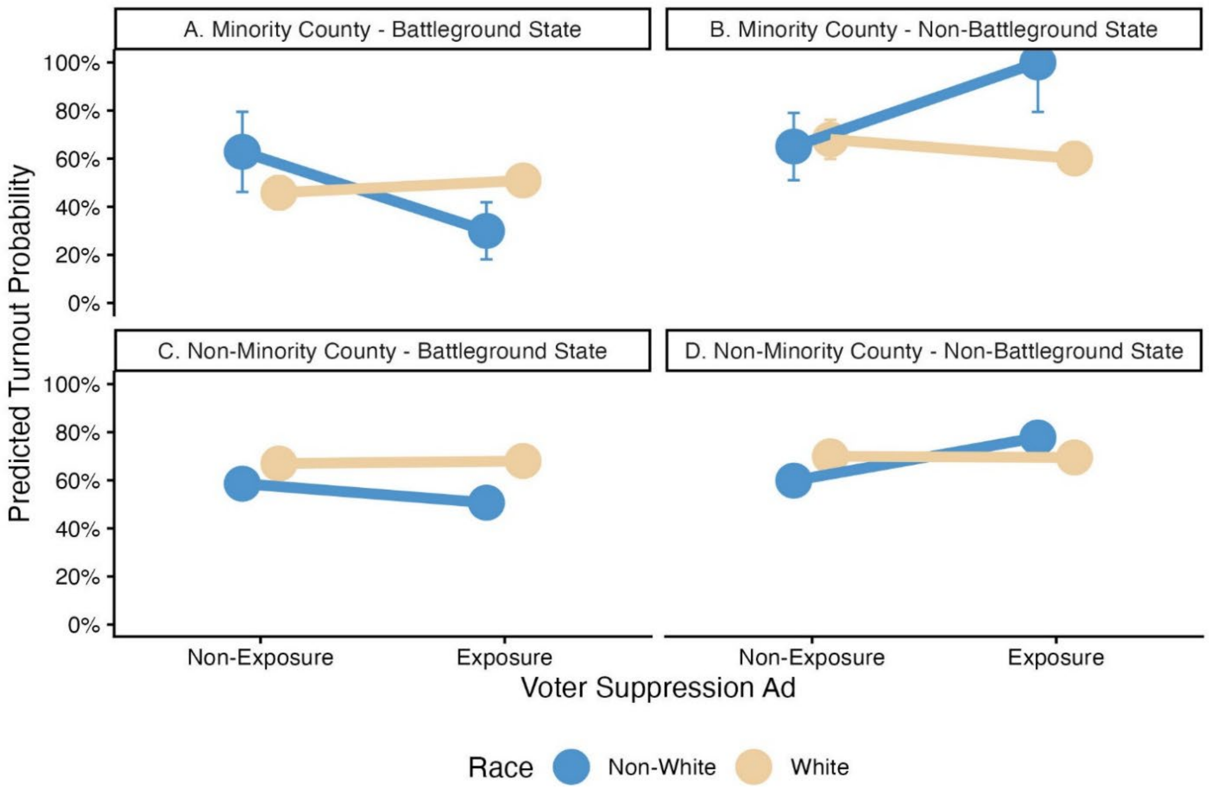

This figure shows predicted turnout probabilities by exposure to Election Boycott type voter suppression ads, comparing white (tan) and non-white (blue) voters across different geographic contexts. Points represent predicted probabilities with 95% confidence intervals from a four-way interaction model examining heterogeneous effects across voter race, battleground state status, and county racial composition. Panel A shows effects for residents of minority-majority counties in battleground states; Panel B shows residents of minority-majority counties in non-battleground states; Panel C represents residents of non-minority counties in battleground states; and Panel D shows residents of non-minority counties in non-battleground states. All models include entropy-balanced weights to account for pre-treatment covariate imbalances between exposed and non-exposed voters. While the average treatment effect of Election Boycott was not statistically significant (b=0.006, SE=0.007, $t(8546)=0.894$, $p=0.371$; $F(1,8546)=0.80$, $R^2<0.001$, RSE=0.106), it suggests statistically significant heterogeneous effects, especially on targeted segments, nonwhites in battlegrounds, and nonwhites in minority counties in battlegrounds, $F(15,8532)=18.63$, $p<0.001$, $R^2=0.032$, RSE=0.105.

B. Deception

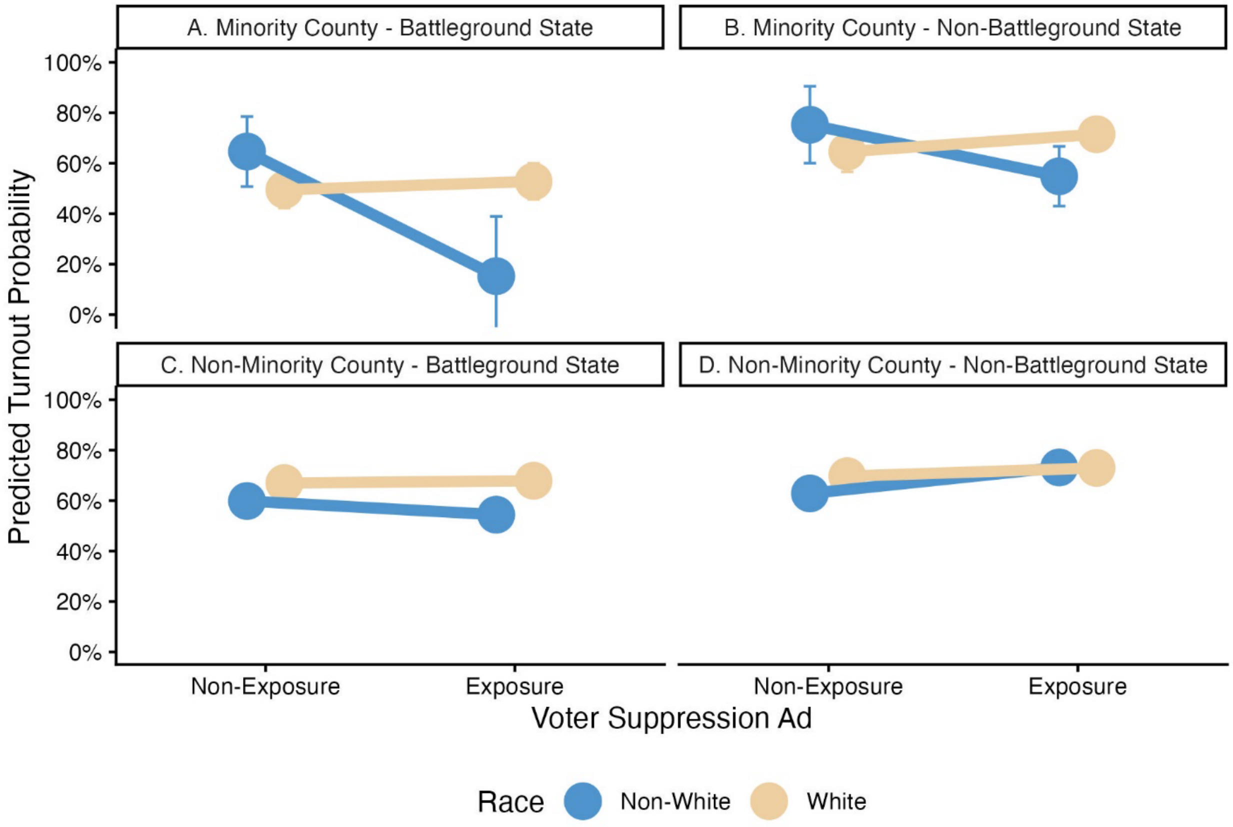

This figure shows predicted turnout probabilities by exposure to Deception-type voter suppression ads, comparing white (tan) and non-white (blue) voters across different geographic contexts. Points represent predicted probabilities with 95% confidence intervals from a four-way interaction model examining heterogeneous effects across voter race, battleground state status, and county racial composition. Panel A shows effects for residents of minority-majority counties in battleground states; Panel B shows residents of minority-majority counties in non-battleground states; Panel C represents residents of non-minority counties in battleground states; and Panel D shows residents of non-minority counties in non-battleground states. All models include entropy-balanced weights to account for pre-treatment covariate imbalances between exposed and non-exposed voters. The model suggests a statistically significant heterogeneous effect of Deception voter suppression exposure, especially on targeted segments, nonwhites in minority counties in battlegrounds, $F(15,8532)=12.99$, $p<0.001$, $R^2=0.022$, RSE=0.121.

The results indicate that election boycott and deception generated similar effects of geo-racially targeted voter suppression---the effects of voter suppression were amplified when voter suppression targeted nonwhites in minority counties, especially in battleground states.

Another type of voter suppression is breaking the coalition of the opposition by attacking the major party candidates with a specific targeting of voters who are on the same ideological side. For example, ads attacking Clinton narrowly target likely Clinton voters. When targeting the "weak link" of the coalition (e.g., those who are not enthusiastic about Clinton, Bernie supporters), it would be more effective; thus, we expected that anti-Clinton voter suppression ads would be amplified among nonwhite voters in minority counties in battlegrounds. As expected, we found anti-Clinton-type voter suppression ads decreased turnout by 3% (ATE, b= -0.030, t (1,

8,546)= -4.34, *p* <.0001). The effects were larger among nonwhites and those in minority counties; nonwhites in minority counties and nonwhites in battlegrounds were most affected by voter suppression, while nonwhites in minority counties of battleground states exhibited the lowest voter turnout rates. All indicate differential effects of geo-racial targeting, even when just examining anti-Clinton-type voter suppression.

C. Anti-Clinton

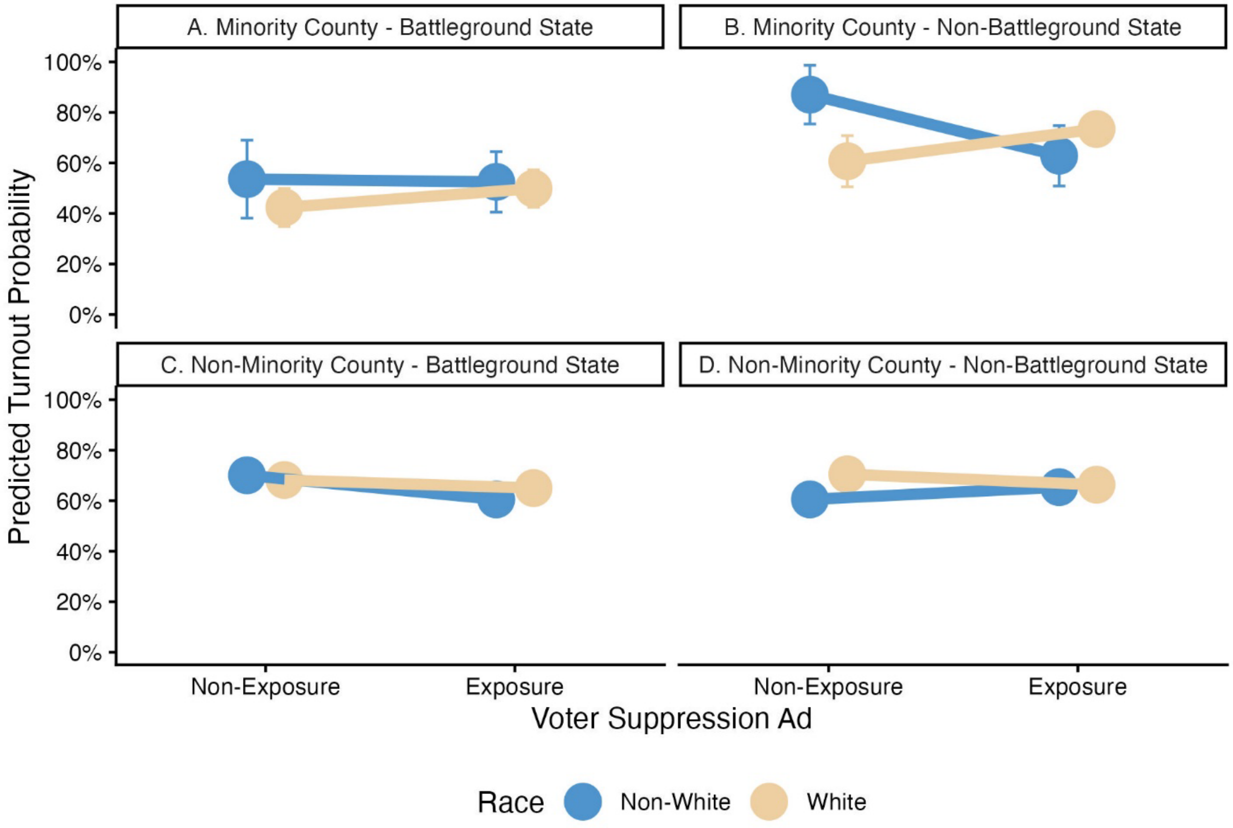

This figure shows predicted turnout probabilities by exposure to Anti-Clinton type voter suppression ads, comparing white (tan) and non-white (blue) voters across different geographic contexts. Points represent predicted probabilities with 95% confidence intervals from a four-way interaction model examining heterogeneous effects across voter race, battleground state status, and county racial composition. Panel A shows effects for residents of minority-majority counties in battleground states; Panel B shows residents of minority-majority counties in non-battleground states; Panel C represents residents of non-minority counties in battleground states; and Panel D shows residents of non-minority counties in non-battleground states. All models include entropy-balanced weights to account for pre-treatment covariate imbalances between exposed and non-exposed voters. The average treatment effect of Anti-Clinton was statistically significant (b=-0.030, SE=0.007, $t(8546)=-4.34$, $p=0.000$. The model indicates statistically significant heterogeneous effects of Anti-Clinton, especially on targeted segments, nonwhites in battlegrounds, and nonwhites in minority counties in battlegrounds, $F(15,8532)=10.02$, $p<0.001$, $R^2=0.017$, RSE=0.173.

D. Pro-Bernie

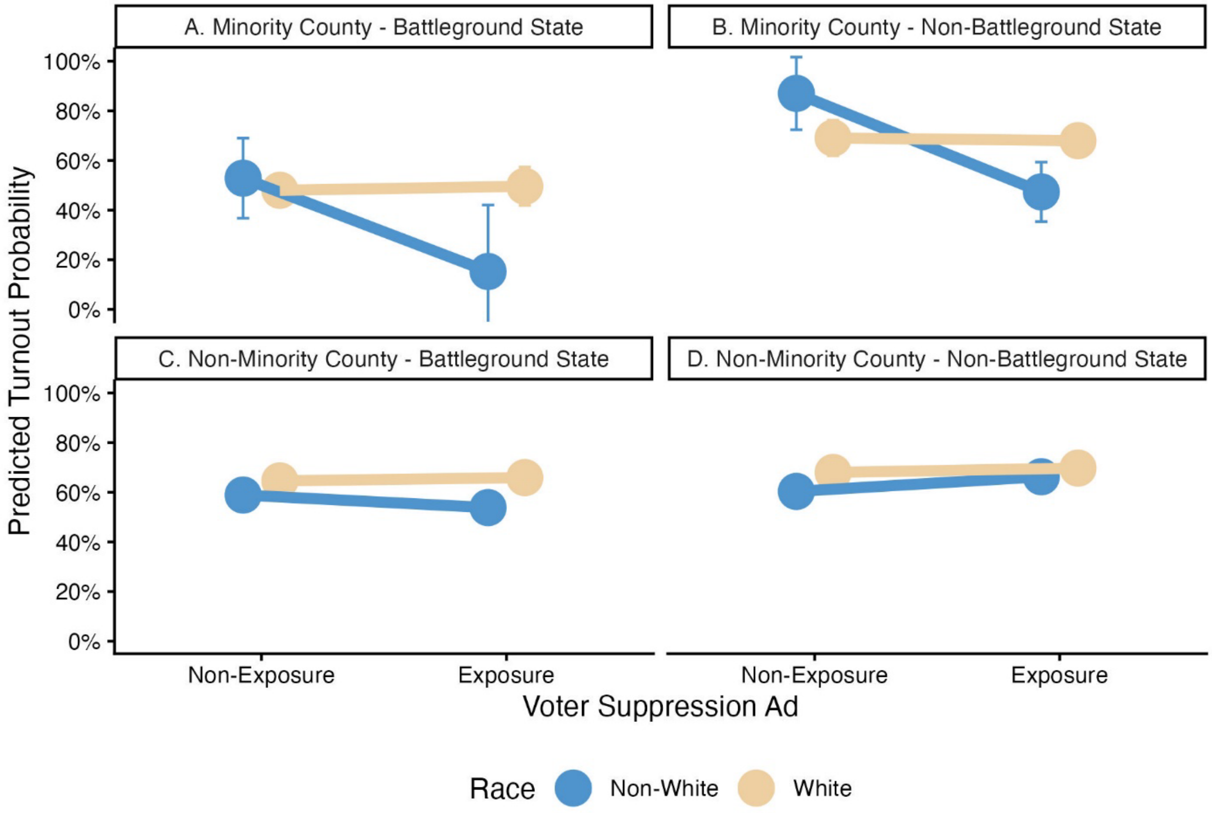

This figure shows predicted turnout probabilities by exposure to Pro-Bernie Sanders type voter suppression ads, comparing white (tan) and non-white (blue) voters across different geographic contexts. Points represent predicted probabilities with 95% confidence intervals from a four-way interaction model examining heterogeneous effects across voter race, battleground state status, and county racial composition. Panel A shows effects for residents of minority-majority counties in battleground states; Panel B shows residents of minority-majority counties in non-battleground states; Panel C represents residents of non-minority counties in battleground states; and Panel D shows residents of non-minority counties in non-battleground states. All models include entropy-balanced weights to account for pre-treatment covariate imbalances between exposed and non-exposed voters. The average treatment effect of Pro-Bernie Sanders was statistically significant (b = .017, SE = .007, $t(8546) = 2.44$, p = .015; $F(1, 8546) = 5.95$, $R^2 < .001$, RSE = .138). The model indicates statistically significant heterogeneous effects of Pro-Bernie Sanders, including stronger effects for targeted segments—nonwhites in battlegrounds (b = -.105, SE = .045, p = .020) and nonwhites in minority counties (b = -.428, SE = .112, p < .001), $F(15, 8532) = 11.09$, p < .001, $R^2 = .019$, RSE = .137.

In a similar vein, pro-Bernie ads targeting likely Clinton voters would also work as voter suppression, as they break the party coalition with a specific targeting of voters who are on the same ideological side. Note, Bernie dropped out from the presidential race earlier primary elections. When targeting Bernie supporters with Bernie support, it would render similar effects as those of anti-Clinton voter suppression ads As expected, the average treatment effect of Pro-Bernie Sanders was statistically significant (b=0.017, SE=0.007, $t(8546)=2.44$, p=0.015; $F(1,8546)=5.95$, $R^2<0.001$, RSE=0.138). The effects were larger among nonwhites in minority counties and nonwhites in battlegrounds, while nonwhites in minority counties of battleground states exhibited the lowest voter turnout rates. All indicate differential effects of geo-racial targeting, even when just examining pro-Bernie ads targeting likely Clinton voters.

4. General Mobilization Effects: The Effects of Get Out The Vote (GOTV) Ads

By contrast, we examined whether and how general mobilization, such as Get Out the Vote (GOTV) ads, influenced voter turnout. By using dictionary terms and phrases often included in GOTV ads (e.g., “Find your poll”; “Vote today”), we identified GOTV ads that provide voting consolidation information for voter turnout mobilization.

We do *not* find any average treatment effect (ATE) of GOTV on turnout (b =0.006, SE = 0.007, t(1, 8546) = 0.833, p = 0.405; F(1,8546) = 0.69, $R^2 < 0.001$, RSE = 0.182). In general, because GOTV ads aim to reach out to the general public as broadly as possible, we expected that any heterogeneous treatment effect would be explained by contextual attributes. We found some targeted GOTV effects concentrated in minority counties, especially among nonwhites in minority counties. This geo-targeted GOTV might have also contributed to the relatively lower turnout rate in minority counties, especially among white voters in minority counties.

Fig.S9: General Mobilization, GOTV, Effects

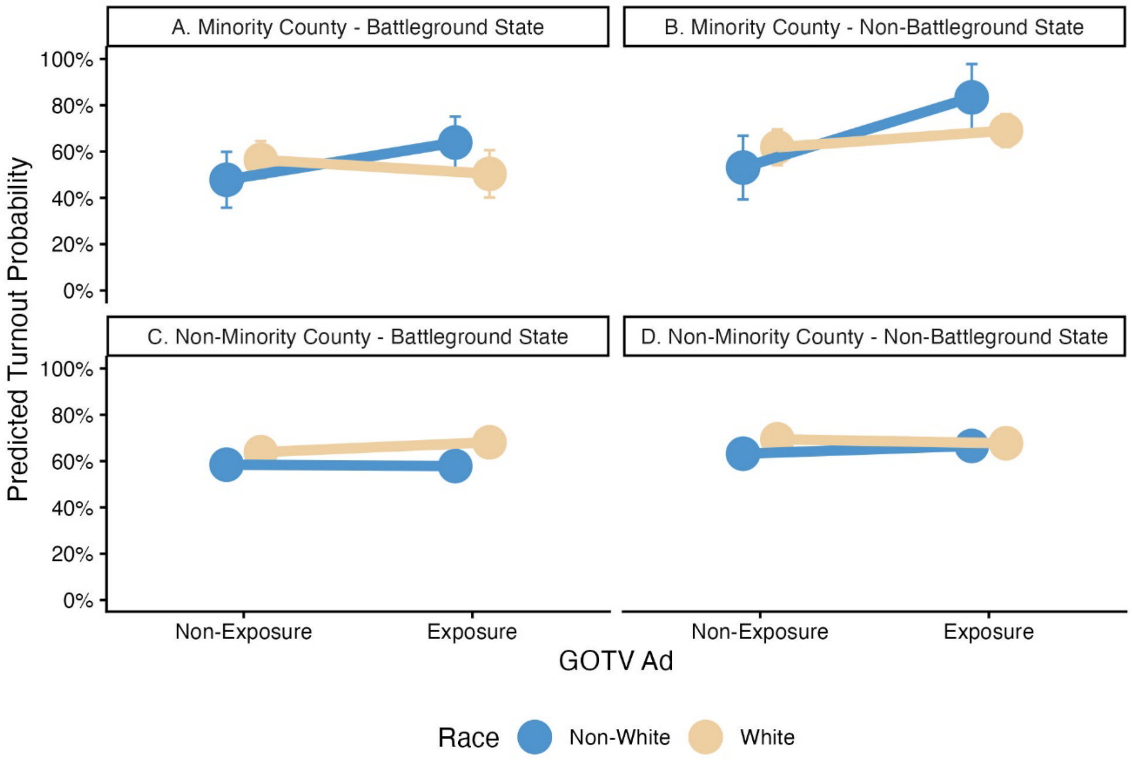

This figure shows predicted turnout probabilities by exposure to GOTV ads, comparing white (tan) and non-white (blue) voters across different geographic contexts. Points represent predicted probabilities with 95% confidence intervals from a four-way interaction model examining heterogeneous effects across voter race, battleground state status, and county racial composition. Panel A shows effects for residents of minority-majority counties in battleground states; Panel B shows residents of minority-majority counties in non-battleground states; Panel C represents residents of non-minority counties in battleground states; and Panel D shows residents of non-minority counties in non-battleground states. All models include entropy-balanced weights to account for pre-treatment covariate imbalances between exposed and non-exposed voters. The ATE shows no significant effect of GOTV ads (b=0.006, SE=0.007, t(8546)=0.833, p=0.405; F(1,8546)=0.69, R^2<0.001, RSE=0.182). (F(15,8532)=7.20, p<0.001, R^2=0.013, RSE=0.181), while suggesting significant heterogeneous effects, F(15,8532)=7.20, p<0.001, R^2=0.013, RSE=0.181, especially in minority counties or battlegrounds.

5. Voter Suppression Effects Conditioned on Timing

We examined the patterns and effects of voter suppression ads across the study period, taking into account the timing of major political events. We first plotted the average cumulative percentage of voter suppression per individual over time to measure both the acceleration and the accumulation of voter suppression ads (**Fig.S10**).

Fig.S10: Average Cumulative Voter Suppression (%) per Person over Time

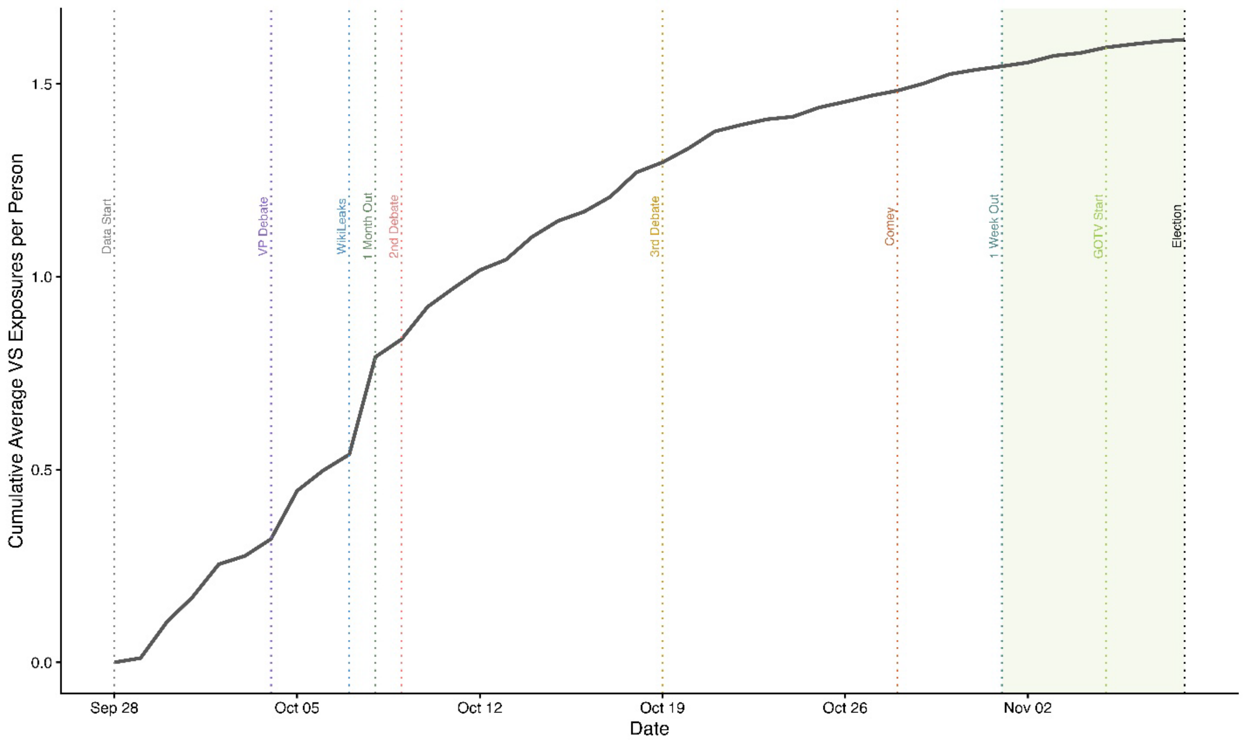

As shown in Fig. S10, voter suppression ads increased dramatically around WikiLeaks, with another sharp spike occurring around the final weekend, a few days before Election Day. Note, the Final Weekend is usually when campaigns' GOTV efforts—including door-to-door canvassing—are concentrated.

To examine whether and how the timing of voter suppression campaigns matters, we also estimated the effects of voter suppression exposure on turnout, conditioned on critical events that occurred during our study period, from the first presidential debate—when data collection began—to Election Day. For temporal analysis, we employed a dynamic treatment framework where exposure timing was defined relative to each critical event, accounting for cumulative exposure. For each temporal cutoff, we implemented separate entropy balancing procedures for each event timing. The balancing procedure follows the standard entropy balancing. The temporal treatment effect estimation was then modeled in the same way as our effect estimates.

Fig.S11: Voter Suppression Effects Conditioned on the Timing of Events

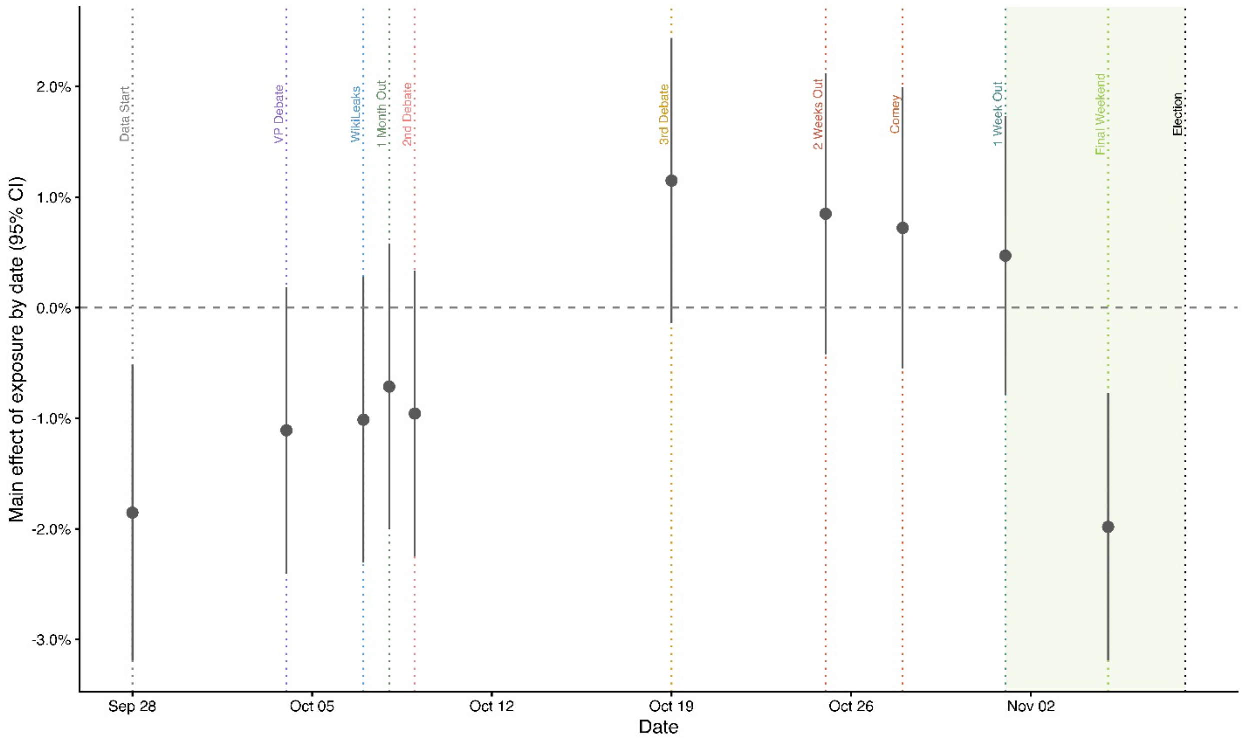

Fig.S11 illustrates voter suppression effects conditioned on the timing of events. The first point estimates (September 28, 2016) indicate the population average treatment effect of voter suppression for the entire study period (thus, same as our reported ATE). A significant voter suppression effect was observed, even when accounting for only a few days of voter suppression ad exposure, specifically between the First Weekend and election day. While effects varied over time, we also observed decreases in voter turnout after the WikiLeaks exposure period, between the WikiLeaks release and the second presidential debate, which corresponded to the increases in voter suppression following WikiLeaks.

Technical Notes: Analysis Techniques

1. PU-Learning

We tracked our participants' turnout records by exactly matching every piece of personally identifiable information (PII) with that of the voter files. See 3.4. Voter Turnout Records (Voter Files) for details about how we tracked and obtained each participant's voting history. We only considered the exact matches as the "matched" cases. A total of 5,995 participants were successfully matched to the voter file. The remaining participants did not contain any records. Voter files only indicate whether an individual voted ($Y_{turnout}$=1) along with "unlabeled" cases. "Unmatched" cases are entirely missing cases. This poses a challenge to us because the unlabeled or unmatched cases indicated either no voter turnout (Y=0) or the cases could be missing for a variety of reasons, such as turnover of GfK, errors in matching, or missing records at the state level. To estimate the voting probability for these unmatched cases, we employed the PU-learning method. This approach involves using the observed characteristics and known voting statuses of the matched cases to train a logistic regression model that predicts the likelihood of voting. Specifically, we used the following variables: age, gender, race, battleground state residence, political party ID, political ideology, income, presidential candidate feeling thermometers, issue importance, and voter registration status. We then calibrated this model to align with the national turnout rate of 61% by applying the corresponding π , to predict voting probabilities for all the participants, including both the matched and unmatched (for details, see 4.5. Voter Turnout: The Individual's Turnout to Vote in **SI**).

Let Y_i for $i = 1, \dots, n$ be the true voting status variable, where $Y_i = 1$ means that the ith person voted and $Y_i = 0$ indicates that they did not vote. Let X_i denote the predictors associated with voting status. Define M_i as the matching indicator, where $M_i = 1$ means that the ith person was matched, and 0 means unmatched. We assume that the matching status M_i does not depend on both the predictors X_i and the true turnout status Y_i, with $P(M_i = 1) = \rho_M$ for all $i = 1, \dots, n$. In the matched sample, we assume that a proportion π of individuals with $Y_i = 1$ is omitted in the voter file, which results in being incorrectly recorded as 0. This misclassification is independent of X_i. Let Z_i denote the observed voting status for matched individuals, $Z = 1, turnout$. Since those who were not labeled as $Y_i = 1$ cannot be confirmed as true voter turnout, we have $P(Z_i = 0 \mid Y_i = 1, M_i = 1) = \pi$ and $P(Z_i = 0 \mid Y_i = 0, M_i = 1) = 1$
We want to learn how X_i and Y_i are related, which we model using logistic regression:

$$P(Y_i = 1 \mid X_i = x_i) = p_\beta(\mathrm{x_i}) = \frac{e^{x_i^\top \beta}}{1+e^{x_i^\top \beta}}.$$

The observed data $\mathcal{D}$ is the union of $(Z_i, x_i, M_i = 1)_{i=1}^{n_m}$ for the matched sample and $(x_i, M_i = 0)_{i=1}^{n_u}$ for the unmatched sample.

The joint observed log-likelihood for this model is

$$\log L(\beta;\mathcal{D}) = \log\left(\prod_{i=1}^{n} p(Z_i, x_i, M_i = 1)^{M_i} p(x_i, M_i = 0)^{1-M_i}\right)$$
$$= \sum_{i=1}^{n_m} \log\{p(Z_i, x_i, M_i = 1)\} + \sum_{i=1}^{n_u} \log\{p(x_i, M_i = 0)\}$$

For the matched sample, we have

$$p(Z_i, x_i, M_i = 1) = p(Z_i | x_i, M_i = 1) P(x_i, M_i = 1)$$

and

$$\begin{aligned} P(Z_i = 1 \mid x_i, M_i = 1) &= \textstyle\sum_{y\in\{0,1\}} P(Z_i = 1, Y_i = y | \, x_i, M_i = 1) \\ &= \textstyle\sum_{y\in\{0,1\}} P(Z_i = 1 \mid Y_i = y, x_i, M_i = 1)\, P(Y_i = y \mid x_i, M_i = 1) \\ &= (1-\pi)\frac{e^{x_i^\top \beta}}{1 + e^{x_i^\top \beta}} \end{aligned}$$

where we use the assumptions that $M \perp (Y, Z) \mid X$, $P(Z = 1 \mid Y = 0) = 1$, and Z ⊥ Y | X.

Note that $P(x_i, M_i = 1)$ does not contain β. Then, the observed log likelihood, incorporating sample weights v_i becomes (up to a proportional constant):

$$\log L(\beta;\mathcal{D}) \propto \textstyle\sum_{i=1}^{n_m} v_i \left\{ z_i \log\left((1-\pi)\frac{e^{x_i^\top \beta}}{1 + e^{x_i^\top \beta}}\right) + (1 - z_i)\log\left(\frac{1 + \pi e^{x_i^\top \beta}}{1 + e^{x_i^\top \beta}}\right) \right\}$$

The parameter β was estimated by maximizing the observed log-likelihood using the EM algorithm. The full log likelihood for the matched data (with sample weights v_i) is

$$\begin{aligned} \sum_{i=1}^{n_m} v_i \log\, p_\beta(z_i, y_i \mid x_i, M_i = 1) &\propto \sum_{i=1}^{n_m} v_i \log\, p_\beta(y_i \mid x_i, M_i = 1) \\ &= \sum_{i=1}^{n_m} v_i \left\{ y_i x_i^\top \beta - \log\left(1 + \exp\,(x_i^\top \beta)\right)\right\} \end{aligned}$$

We also have $E[Y_i \mid Z_i, x_i, M_i = 1] = E[Y_i \mid Z_i, x_i] = 1$ if $Z_i = 1$ and when $Z_i = 0$,

$$E[Y_i \mid Z_i = 0, x_i] = P(Y_i = 1 \mid Z_i = 0, x_i) = \frac{P(Y_i = 1, Z_i = 0 \mid x_i)}{P(Z_i = 0 \mid x_i)} = \frac{\pi e^{x_i^\top \beta}}{1 + \pi e^{x_i^\top \beta}}$$

The EM Algorithm is as follows:

Input: an initialization β^0
for $m = 0,1, \ldots$ do

1 **E-step** : estimate y_i at $\beta = \beta^m$ by $\hat{y}_i(\beta^m) = \left(\frac{\pi e^{x_i^\top \beta^m}}{1+\pi e^{x_i^\top \beta^m}}\right)^{1-z_i}$

2 **M-step** : obtain β^{m+1} by

$$\beta^{m+1} \in \underset{\beta}{\operatorname{argmax}} \left\{ \sum_{i=1}^{n_m} v_i \left(\hat{y}_i(\beta^m)(x_i^\top \beta) - \log\left(1 + e^{x_i^\top \beta}\right) \right) \right\}$$

The algorithm contains a hyperparameter π. To specify this hyperparameter, we searched over values of π to find the value that produces aggregate turnout (the mean of the predicted probabilities of voting) that most closely approximates the actual national turnout rates using root-mean-squared error.

Finally, given the misclassification rate π and estimated model parameter $\hat{\beta}$, the turnout probability for each individual was estimated as follows.

For the matched sample, the estimated turnout probability is given by:

$$\hat{Y}_i = \hat{E}[Y_i | x_i, z_i] = \left(\frac{\pi e^{x_i^\top \hat{\beta}}}{1 + \pi e^{x_i^\top \hat{\beta}}}\right)^{1-z_i}$$

Note that if $z_i = 1$, then the individual is an individual who is confirmed to have voted, and $\hat{Y}_i =$ 1; otherwise, the probability of turnout is estimated using the fitted model and adjusted by the misclassification rate π.

For the unmatched sample, turnout probability is estimated by

$$\hat{Y}_i = \hat{E}[Y_i | x_i] = \left(\frac{e^{x_i^\top \hat{\beta}}}{1 + e^{x_i^\top \hat{\beta}}}\right)$$

We then searched over values of π to find the value that produced aggregate turnout (the mean of the predicted probabilities of voting) that closely approximates the actual national turnout rates using root-mean-squared error. We then used the π value that best approximated actual turnout and used it to estimate the predicted probability of turning out to vote for all participants (both matched and unmatched). We used this probability as the mean dependent variable for voter turnout.

2. Identification and Balance Strategies for Causal Analysis

To estimate the causal effects of voter suppression ads on voter turnout, we employed a quasi-experimental approach. We examined the (weighted) differences between the "treatment group," (X; exposure to voter suppression ads where Exposure = 1, No Exposure = 0) and "control group" or "counterfactual," (no exposure to voter suppression ads), in terms of predicted probability of voter turnout while the distributions of the covariates being the same across the treatment and control groups. The unit of analysis here is an individual, and the outcome variable is an individual's predicted turnout probability (Y: Turnout =1 ~ No turnout = 0).

The causal inference protocol employed in this study involves three principal steps: selection of covariates, entropy balancing, and treatment effect estimation.

Selection of Covariates: The selection of covariates was primarily theory-driven. Drawing upon electoral behavior literature and political campaign research, we included variables that are correlated with our dependent variable, voter turnout (e.g., prior turnout history, gender, race, income, education, age, and so forth) or with the treatment, i.e., voter suppression ad exposure (e.g., candidate feeling thermometers, issue importance, political ad exposure, total ad exposure). We also used Double Lasso Selection as a reference point to specify which covariates should not be excluded as covariates in later balancing processes for causal inferences. This method addresses potential omitted variable bias and enhances the robustness of our statistical inferences. Specifically, we model two distinct outcomes from our survey data: the independent variable of interest, exposure to a voter suppression ads ($y_1 = \text{VSExposure}$) and the dependent variable of interest, voter turnout ($y_2 = \text{Turnout}$).

For each outcome, we conducted Lasso regressions. First, we selected relevant variables for VSExposure by minimizing the following objective function using logistic regression with an ℓ_1-penalty:

$$(1)\ L(\beta_1) = -\sum v_i\{\, y_{1i}\, x_i^{\top}\beta_1 - \log(1+\exp(x_i^{\top}\beta_1))\} + \lambda_1 \sum_{j=1}^{p} |\beta_j|$$

where v_i denotes the sample weights, and predictor x_i includes a variety of potential covariates observed in our data. We determined the optimal penalty parameter (λ_1) through cross-validation to minimize prediction error, selecting significant predictors that contribute to understanding VSExposure. Similarly, for voter turnout, we performed the following linear regression with an ℓ_1-penalty:

$$(2)\ L(\beta_2) = \sum_{i=1}^{n} v_i\, (y_{2i} - x_i^{\top}\beta_2)^2 + \lambda_2 \sum_{j=1}^{p} |\beta_j|$$

where we also optimized λ_2 via cross-validation. Following these individual regressions, we intersected the sets of predictors deemed significant in both models ($\hat{\beta}_1 \neq 0$ and $\hat{\beta}_2 \neq 0$), retaining the variables influencing both voter suppression exposure and turnout as covariates. In subsequent entropy balancing, however, we added theoretical predictors for voter turnout or voter suppression ad exposure, in addition to selected covariates from double lasso models.

Entropy Balancing. Next, we continued to employ entropy balancing. Entropy balancing is a pre-processing technique that adjusts the weights of observations in the control group to

achieve a balance in covariate distributions between the treatment and control groups, thereby approximating a randomized experimental setting. This method, first introduced by Hainmueller in 2012, systematically reweights the control group to equalize the covariate distributions with the treatment group.

In our study, entropy balancing was implemented following the identification of key covariates as described in the previous section. We focused on achieving balance across these critical covariates to ensure that any subsequent analysis of treatment effects would not be confounded by baseline differences. The mathematical foundation of this approach is given by the following optimization problem:

(3)

$$\text{minimize} \quad \sum_{i \in C} w_i \log\left(\frac{w_i}{v_i}\right)$$

(4) Subject to the constraints:

$$\sum_{i \in C} w_i = 1, \quad \sum_{i \in C} w_i x_{ki} = \bar{x}_{kT}, \quad k = 1, \ldots, K$$

where w_i represents the weights assigned to each control unit i, v_i denotes the base weights (initial sampling weights or demographic adjustments. In our case, poststratification with cell weights benchmarking on the Census), x_{ki} are the selected covariates, and $\bar{x}_{kT}$ are the corresponding means of these covariates in the treatment group. This formulation ensures that the reweighted control group exactly matches the treatment group in terms of the specified covariate means, thus optimizing the balance between the groups.

The entropy balancing method applies these weights through an iterative algorithm that adjusts w_i to satisfy the balance constraints, effectively standardizing the treatment and control groups with respect to the included covariates. In our application, the covariate matrix $X_{\text{intersect}}$ includes the variables identified in the double selection process. By achieving covariate balance, this method mitigates the risk of omitted variable bias and enhances the causal interpretation of the observed relationships.

Upon computing the entropy balanced weights, these are integrated into our dataset: control group members (those not exposed to a voter suppression ad) had their weight replaced with their entropy balance weights; the treatment group (those exposed to voter suppression ads) retained their original weights. This use of the entropy balancing weights ensures covariate balance between the control (those not exposed to voter suppression ads) and the treatment group (those exposed to voter suppression ads).

To assess the robustness of the entropy balancing model, we conducted both convergence tests and sensitivity analysis. First, we assessed the balance, i.e., convergence of the mean values of the covariates between the treatment and control groups after the entropy balancing processes. Second, we conducted a sensitivity analysis to quantify potential bias due to unobserved or unmeasured confounders.

Convergence Test. First, to assess the effectiveness of our entropy balancing procedure in achieving covariate balance between the treatment and control groups, we analyzed the standardized mean differences (SMDs) for each covariate before and after applying the entropy balancing weights. The closer these values after the balancing are to zero, the better the balance

achieved by the entropy balancing. As shown in Fig.S2 in **SI**, we were able to achieve nearly perfect covariate balance after the entropy balancing.

Sensitivity Analysis. Sensitivity analysis examines the extent to which unobserved confounders could confound the relationship between the treatment effect of exposure to voter suppression advertisements on voter turnout. We systematically varied two parameters: the effects of a potential unmeasured confounder on the probability of turnout (γ) and the effects of receiving voter suppression ads on this confounder (α). By calculating the product of these parameters ($\gamma \times \alpha$), we estimate the potential bias introduced in our treatment effect estimation.

We created a grid of values for γ and α, ranging from 0 to 2 in increments of 0.01, and compute the corresponding bias for each combination. This matrix of biases allows us to visualize the range of possible biases and understand at what levels of γ and α our conclusions about the treatment effect would be invalidated. Using a linear regression model fitted with the actual covariates from our study, we adjusted the estimated treatment effect by the calculated biases to obtain a range of potential outcomes under different scenarios of unmeasured confounding. We plotted these adjusted treatment effects against our bias parameters to visually assess the sensitivity of our results. This plot (Fig.S6 in **SI**) includes lines representing statistical significance thresholds, allowing us to determine which values of γ and α would lead to different conclusions about the effectiveness of the voter suppression ads. As seen in Fig.S6, we find that our model is robust to potential confounding variables where a potential confounder would have to confound the relationship between treatment and effect more than any of the observed variables we include in our model. It is highly unlikely that causal effects are explained by unobserved or unmeasured confounders.

*Effects Assessments: ATE and HTE*s. Finally, to quantify the impact of exposure to voter suppression ads on voter turnout, we initially computed the Average Treatment Effect (ATE) using a linear regression model with entropy balancing weights applied. This model assesses the relationship between predicted voter turnout and ad exposure while controlling for balanced covariates ensured by the entropy balancing procedure.

We specify the model as follows:

(5) $$\text{Turnout} = \beta_0 + \beta_1 \cdot \text{VSExposure} + \epsilon, \quad \text{weighted by } w_i$$

Where w_i represents the entropy balancing weights applied to each observation in the regression to adjust for any covariate imbalances between the treatment and control groups. **Fig.3** in **Main Text** presents the results

To explore a potential Heterogeneous Treatment Effect (HTE), we further examined how this relationship varies across different subgroups of the population. Specifically, we focused on three key moderating variables: nonwhite, battleground state, and minority county. For each moderating variable, we divided the main dataset into corresponding subgroups based on the binary classification of the variable (e.g., nonwhite and white individuals). We then repeated the entropy balancing process separately for each group to ensure that within each subgroup, the covariates would be balanced as much as possible. After rebalancing, we merged the subgroups and introduced interaction terms between the moderating variables and the main treatment variable in the linear model to estimate the HTEs. **Fig.4** in **Main Text** presents the results.

We also included multi-way interaction terms (up to full four-way interactions) in our model based on the original, unsplit, entropy-balanced dataset. This comprehensive modeling helped us capture complex interaction effects that might be obscured in simpler models.

Additionally, for a more straightforward interpretation of how ad exposure affects different groups, we calculated and compared the simple group means for all combinations of the moderating variables using the main entropy balanced dataset. This method provides a direct, though less statistically refined, insight into the varying impacts across different demographic and geographic segments.

This structured approach to analyzing treatment effects ensures a thorough exploration of both the average and variable impacts of voter suppression ads, highlighting the importance of context in understanding voter behavior.

Treatment Effects with Different Control Groups (Counterfactuals). To check robustness, we also conducted the same entropy balancing strategies and the same covariates (where appropriate) with different control groups. Given that we do not have a true counterfactual in reality, testing the voter suppression effects (treatment effects) with differently operationalized control groups or subgroups would be a reasonable way to examine how robust the estimated treatment effects would be. See the results in SI.

Placebo Tests. We also employed two placebo tests, a) false shock (i.e., false treatment replacing true treatment) and b) prediction for the 2012 presidential turnout (i.e., false outcome and false time order, replacing the 2016 presidential voter turnout. In both tests, we employed the same entropy balancing strategies, using the same covariates (where applicable).

Replications of Causal Analysis with Different Matching and Balancing Strategies. In addition to entropy balancing, we also replicated the same procedure with different identification, matching, and balancing strategies: a) Exact matching and b) Full matching with covariate balance propensity scores (CBPS). Exact matching is a stringent method used in causal inference to ensure that each treated unit is compared to one or more control units that are identical on all observed covariates. This method directly addresses confounding by eliminating differences between treated and control groups in the covariates used for matching. By ensuring an exact match on all included covariates, this approach creates pairs or groups of units where the only systematic difference is the treatment itself, thus providing a robust basis for estimating causal effects.

Mathematically, exact matching can be described as follows:

For a given treated unit with covariates X_t, control units are selected such that their covariates X_c satisfy the condition $X_t = X_c$.

This process is repeated across all treated units, ensuring that each treated unit is paired only with control units that have identical covariate values. As a result, the average treatment effect (ATE) can be estimated by comparing outcomes between these exactly matched groups, effectively controlling for all observed confounding variables. In our analysis, we matched on the same covariates used throughout the manuscript.

Full matching is a method of matching in causal inference that creates pairs or clusters of treated and control units, where each cluster is formed to minimize overall differences across a set of covariates. Unlike exact matching, full matching does not require an exact match on

covariates but rather optimizes the balance of covariates across treated and control groups as a whole. This method is particularly useful in settings where exact matches are rare due to the high dimensionality of data or when continuous variables are involved. In full matching, each treated unit can be matched to one or more control units, or vice versa, forming variable-sized matched sets that collectively cover the entire sample. The goal is to minimize the average distance across all matches, typically measured using a propensity score or other distance metrics. The optimization is generally achieved through a combination of greedy algorithms and optimization techniques.

Mathematically, full matching solves the following optimization problem:

Minimize

$$\sum_{i \in T, j \in C} d(X_i, X_j) \cdot I_{ij}$$

Where $d(X_i, X_j)$ represents a distance measure between the covariates of treated group i and control group j, and I_{ij} is an indicator function that is 1 if units i and j are matched and 0 otherwise. The distance can be computed using methods such as the generalized linear model (GLM) or the covariate balancing propensity score (CBPS), which offer different approaches for assessing and controlling for covariate imbalances. In this study, we used CBPS. CBPS enhances the traditional propensity score approach by ensuring that the propensity score model is specified so that it simultaneously balances the covariates across treatment groups. This method adjusts the propensity score estimation process to optimize the balance of covariates, potentially making it more robust to model misspecification compared to GLM.

Survey Questionnaire

*Items are presented **in the same order** as in the survey questionnaire used for this study

* The survey items are used as **covariates (control variables)** in the hierarchical linear/nonlinear model (HLM) of targeting patterns and as **covariates** to balance (i.e., covariate adjustments) the control group (nonexposure group, voter suppression exposure =0)) with entropy balancing, which makes the distributions of covariates as the same as the treatment group (exposure group, voter suppression exposure =1). Race (nonwhite=1) was also used as a moderator in the Heterogeneous Treatment Effect (HTE) estimate models.

* For more details, including recoding and descriptive statistics, see section 4.3 in **SI**.

First, we would like to know a little bit about you.

[**Gender**]
Are you male or female?
Male; Female

[**Age**]
Please enter your birth month and year below:
Birth month:
Birth year:

[**Race**]
Which of the following best describes your race?
Caucasian; African-American; Latino/Hispanic; Asian; Other: Specify

[**Education**]
What is the highest level of schooling you have completed?
8th grade or less; Some high school; High school graduate; Some college or Associate Degree; Bachelor's degree; Some post-secondary education; Graduate or professional degree

[**Income**]
The next question is about the total income of YOUR HOUSEHOLD for the PAST 12 MONTHS. Please include your income PLUS the income of all members living in your household (including cohabiting partners and armed forces members living at home). Please count income BEFORE TAXES and from all sources (such as wages, salaries, tips, net income from a business, interest, dividends, child support, alimony, and Social Security, public assistance, pensions, or retirement benefits).
What was your HOUSEHOLD income (before taxes) in the past 12 months?
Under $20,000; $20,000-$39,999; $40,000-$59,999; $60,000-$79,999; $80,000-$119,999; $120,000 to 249,999; $250,000 or more

[**Issue Importance**]
Recently, there has been a lot of discussion about certain social and political issues. How important is each of the following issues to YOU PERSONALLY? [the order of the issues is randomized]
Extremely unimportant 1 2 3 4 5 6 7 Extremely important
Abortion
Climate change
College affordability
Gay marriage
Global Terrorism

Gun control
Health care
Immigration
International trade
Jobs/employment
Minimum wage
Race relations
Social security
Tax cuts

[**Political Ideology**]
We hear a lot of talk these days about liberals and conservatives. When it comes to politics, do you usually think of yourself as extremely liberal, liberal, slightly liberal, moderate or middle of the road, slightly conservative, extremely conservative?
Extremely liberal; Liberal; Slightly liberal; Moderate or middle of the road; Slightly conservative; Conservative; Extremely conservative

[**Party ID**]
Given the following choices, which best represents your political party affiliation?
Democrat; Independent; Republican; Other: Specify

[**Voter Registration**]
Are you registered to vote in this election?
Yes; No

[**State**]
If yes to [Voter Registration]: In which state are you currently registered to vote? (drop down menu listing all the states including District of Columbia and Puerto Rico in alphabetical order)
If no to [Voter Registration]: In which state are you able to register to vote?

[**Vote 2012**, self-reported]
***Notes**: For the placebo tests we employed, we used actual turnout records (voter turnout history from voter files), not this item. *This item is used for covariate adjustment only (to reduce missing cases).
In 2012, the major candidates for president were Mitt Romney for the Republicans and Barack Obama for the Democrats. In that election, did you definitely vote, definitely not vote, or are you not completely sure whether you voted?
Definitely DID vote; Definitely DID NOT vote; Not completely sure

[**Feeling Thermometers**]
We would like to get your feelings towards the current presidential candidates using a feeling thermometer. Please indicate how you feel about each candidate, where ratings between 0 degrees and 50 degrees mean that you don't feel favorable toward the person, and ratings between 50 degrees and 100 degrees mean that you do feel favorable toward the person. You would rate the person at the 50 degree mark if you don't feel particularly warm or cold toward the person. Please indicate how you feel about each of the following candidates [the order of candidates is randomized]
[Continuous slide bar is provided for each candidate, with 0, 50, 100 anchoring indicators]
Donald Trump
Hillary Clinton
Jill Stein
Gary Johnson

References

1. Aral, S. & Eckles, D. Protecting elections from social media manipulation | Science. **365**, (2019).

2. Zhou, A., Metaxa, D., Kim, Y. M. & Jaidka, K. User-Centric Behavioral Tracking: Lessons from Three Case Studies with Do-It-Yourself Computational Pipelines. *J. Advert.* **53**, 791–809 (2024).

3. Dahlgaard, J. O., Hansen, J. H., Hansen, K. M. & Bhatti, Y. Bias in Self-reported Voting and How it Distorts Turnout Models: Disentangling Nonresponse Bias and Overreporting Among Danish Voters. *Polit. Anal.* **27**, 590–598 (2019).

4. Meta, F. Meta for Business. *Meta for Business* https://www.facebook.com/business/news/How-Facebook-Ads-Work.

5. About custom audiences. *Meta Business Help Center* https://www.facebook.com/business/help/744354708981227.

6. About Lookalike Audiences. *Meta Business Help Center* https://www.facebook.com/business/help/164749007013531.

7. Meta for Business. *Meta for Business* https://www.facebook.com/business/news/Core-Audiences.

8. Senate Select Committee on Intelligence. Publications | Intelligence Committee. https://www.intelligence.senate.gov/publications/report-select-committee-intelligence-united-states-senate-russian-active-measures.

9. Valliant, R., Dever, J. A. & Kreuter, F. *Practical Tools for Designing and Weighting Survey Samples*. (Springer, 2018).

10. Wang, T. A. *The Politics of Voter Suppression: Defending and Expanding Americans' Right to Vote*. (Cornell University Press, Ithaca, 2012).

11. Kim, Y. M. *Uncover: Strategies and Tactics of Russian Interference in US Elections Russian Groups Interfered in Elections with Sophisticated Digital Campaign Strategies*. (2018).

12. Krippendorff, K. *Content Analysis: An Introduction to Its Methodology*. (SAGE Publications, Inc, Los Angeles London New Delhi Singapore, 2013).

13. Hainmueller, J. Entropy Balancing for Causal Effects: A Multivariate Reweighting Method to Produce Balanced Samples in Observational Studies. *Polit. Anal.* **20**, 25–46 (2012).

14. Holbrook, A. L. & Krosnick, J. A. Social desirability bias in voter turnout reports: Tests using the item count technique. *Public Opin. Q.* **74**, 37–67 (2010).

15. Song, H. & Raskutti, G. PUlasso: High-Dimensional Variable Selection With Presence-Only Data. *J. Am. Stat. Assoc.* **115**, 334–347 (2020).

16. Raudenbush, S. W. & Bryk, A. S. *Hierarchical Linear Models: Applications and Data Analysis Methods (Advanced Quantitative Techniques in the Social Sciences)*. (SAGE Publications, Inc, 2001).

17. Lewis-Beck, M., Bryman, A. & Futing Liao, T. Hierarchical (NON)Linear Model. in *The SAGE Encyclopedia of Social Science Research Methods* pages 461-461.

18. Belloni, A., Chernozhukov, V. & Hansen, C. Inference on Treatment Effects after Selection among High-Dimensional Controls. *Rev. Econ. Stud.* **81**, 608–650 (2014).

19. Urminsky, O., Hansen, C. & Chernozhukov, V. Using Double-Lasso Regression for Principled Variable Selection. SSRN Scholarly Paper at https://doi.org/10.2139/ssrn.2733374 (2016).

20. Thoemmes, F. J. & Kim, E. S. A systematic review of propensity score methods in the social sciences. *Multivar. Behav. Res.* **46**, 90–118 (2011).

21. King, G. & Nielsen, R. Why Propensity Scores Should Not Be Used for Matching. *Polit. Anal.* **27**, 435–454 (2019).

22. Zhao, Q. & Percival, D. Entropy Balancing is Doubly Robust. *J. Causal Inference* **5**, (2017).

23. Hainmueller, J., Liu, J., Liu, Z., Mummolo, J. & Xu, Y. A Response to Recent Critiques of Hainmueller, Mummolo and Xu (2019) on Estimating Conditional Relationships. Preprint at https://doi.org/10.48550/arXiv.2502.05717 (2025).

24. Simonsohn, U. Interacting With Curves: How to Validly Test and Probe Interactions in the Real (Nonlinear) World. *Adv. Methods Pract. Psychol. Sci.* **7**, 25152459231207787 (2024).

25. Cinelli, C. & Hazlett, C. Making Sense of Sensitivity: Extending Omitted Variable Bias. *J. R. Stat. Soc. Ser. B Stat. Methodol.* **82**, 39–67 (2020).

26. Thabane, L. *et al.* A tutorial on sensitivity analyses in clinical trials: the what, why, when and how. *BMC Med. Res. Methodol.* **13**, 92 (2013).

27. Stuart, E. A. Matching methods for causal inference: A review and a look forward. *Stat. Sci. Rev. J. Inst. Math. Stat.* **25**, 1 (2010).

28. Ho, D. E., Imai, K., King, G. & Stuart, E. A. Matching as nonparametric preprocessing for reducing model dependence in parametric causal inference. *Polit. Anal.* **15**, 199–236 (2007).

29. Hansen, B. B. Full matching in an observational study of coaching for the SAT. *J. Am. Stat. Assoc.* **99**, 609–618 (2004).
